# Acute respiratory distress vs healthy lung environments differently affect mesenchymal stromal cell extracellular vesicle miRNAs

**DOI:** 10.1016/j.jcyt.2025.01.006

**Published:** 2025-01-20

**Authors:** Sara Rolandsson Enes, Irakli Dzneladze, Thomas H. Hampton, Samuel L. Neff, Lori Asarian, Jayita Barua, Tobias Tertel, Bernd Giebel, Nicolas Pereyra, David H. McKenna, Pingzhao Hu, Erica Acton, Alix Ashare, Kathleen D. Liu, Anna D. Krasnodembskaya, Karen English, Bruce A. Stanton, Patricia R.M. Rocco, Michael A. Matthay, Claudia C. dos Santos, Daniel J. Weiss

**Affiliations:** 1 Department of Medicine, Larner College of Medicine, University of Vermont, Burlington, Vermont, USA; 2 Department of Experimental Medical Science, Faculty of Medicine, Lund University, Lund, Sweden; 3 Interdepartmental Division of Critical Care, Department of Medicine and the Keenan Center for Biomedical Research, St. Michael’s Hospital, University of Toronto, Toronto, Canada; 4 Department of Microbiology and Immunology, Geisel School of Medicine at Dartmouth, Hanover, New Hampshire, USA; 5 Institute of Transfusion Medicine, University Hospital Essen, University of Duisburg-Essen, Essen Germany; 6 Department of Biochemistry and Molecular Biology, The University of British Columbia, Canada, The University of British Columbia Centre for Blood Research, Vancouver, Canada; 7 Department of Laboratory Medicine and Pathology, University of Minnesota, Minneapolis, USA; 8 Department of Biochemistry and Medical Genetics, University of Manitoba, Winnipeg, Canada; 9 Section of Pulmonary and Critical Care Medicine, Dartmouth-Hitchcock Medical Center, Lebanon, New Hampshire, USA; 10 Departments of Medicine and Anesthesiology and the Cardiovascular Research Institute, University of California San Francisco; 11 Wellcome-Wolfson Institute for Experimental Medicine, School of Medicine, Dentistry, and Biomedical Sciences, Queens University, Belfast, UK; 12 Cellular Immunology Laboratory, Biology Department, Kathleen Lonsdale Institute for Human Health Research, Maynooth University, Maynooth, Co. Kildare, Ireland; 13 Laboratory of Pulmonary Investigation, Carlos Chagas Filho Institute of Biophysics, Federal University of Rio de Janeiro; 14 National Institute of Science and Technology for Regenerative Medicine, Rio de Janeiro, Brazil

**Keywords:** acute respiratory distress syndrome, bronchoalveolar lavage fluid, CFTR, exosomes, extracellular vesicles, mesenchymal stromal cells, miRNA

## Abstract

The acute respiratory distress syndrome (ARDS) inflammatory environment alters mesenchymal stromal cell (MSC) gene and protein expression but effects on micro-RNA (miRNA) content of MSC-extracellular vesicle (EVs) remain unknown. To assess this, sequencing analysis of EV-miRNAs prepared from human bone marrow-derived MSCs (hMSCs) exposed *ex vivo* to bronchoalveolar lavage fluid (BALF) from ARDS patients or healthy volunteers (HV) identified a number of differentially expressed miRNAs. Discriminant, differential expression, and functional enrichment analyses identified 14 miRNAs significantly changed following ARDS versus HV BALF exposure. Network analysis showed 4 (miR-760, miR-3175, miR-885-3p, and miR-766-3p) of the 14 EV-miRNAs formed a regulatory “hub”, suggesting co-targeting of specific gene pathways. *In silico* prediction identified a number of pathways important in lung injury. Two miRNAs involved in regulation of the cystic fibrosis transmembrane conductance regulator (CFTR), miRNA-145-5p and miRNA-138-5p, were also significantly increased in ARDS BALF-exposed hMSCs EVs. Functionally, EVs from hMSCs exposed to either ARDS or HV BALF had differential effects on CFTR Cl^−^ secretion by cultured primary human bronchial epithelial cells, an effect predicted to reduce mucociliary clearance. The potential clinical impact of these finding highlights the need for further studies assessing the role of hMSC-EV miRNAs in regulating lung inflammation and mucociliary clearance.

## Introduction

Cell-based therapies utilizing mesenchymal stromal cells (MSCs) are being increasingly investigated for potential use as treatment for acute respiratory distress syndrome (ARDS), including ARDS resulting from SARS-CoV-2 infection (COVID-19). These approaches are based on the paracrine actions which can contribute to reduction of lung inflammation, facilitation of endothelial repair, increased alveolar fluid clearance, and regulation of lung epithelial and endothelial permeability [1–3]. MSC-derived extracellular vesicles (EV) preparations contain cytokines, growth factors, and signaling lipids as well as mRNAs and miRNAs that may be the effectors of these beneficial actions [4,5]. Preclinical investigations in models of acute lung injury have consistently demonstrated that both systemic or intratracheal MSC administration, and more recently systemic administration of MSC-EVs, result in amelioration of lung injury [1–4]. These have provided a basis for initial clinical investigations of MSC-based therapies in both non-COVID and COVID-related ARDS [6–13].

These clinical trials to date have uniformly demonstrated safety, however, they have not yet consistently demonstrated efficacy. Although recent meta-analyses demonstrate an overall trend towards efficacy [14–16] and a secondary analysis of one double blind, randomized clinical trial reported evidence of favorable biologic effects on levels of inflammatory mediators in the bronchoalveolar lavage fluid (BALF) comparing patients who received MSCs versus placebo [17]. While many factors may be responsible for the lack of improved outcomes in the different studies, there remains a fundamental lack of knowledge as to the fate and actions of administered MSCs in the diseased human lung microenvironment. In part through expression of toll-like and other cell surface receptors, MSCs react to environmental factors and accordingly alter anti-inflammatory actions including their secretome, which can alter the impact of applied MSCs to their environment, and the viability of the infused cells[18–21]. For example, *ex vivo* exposure to BALF or serum samples from ARDS or cystic fibrosis patients, used as surrogates for the *in vivo* inflammatory environment, has a significant impact on the composition of the MSC secretome and the function on their target cells, especially on macrophages by enhancing their anti-inflammatory actions [22–24]. Accordingly, it appears very likely that the inflammatory *in vivo* environment encountered in ARDS significantly affects the therapeutic efficacy of applied MSCs, at least in parts executed by the MSCs’ secretome including their secreted EVs.

Therefore, as the role of EV miRNAs and their specific associated contents remains incompletely understood [25,26], the aim of the current study was to determine the effects of human MSC (hMSC) exposure to human ARDS or healthy volunteer (HV) BALF on the composition and potential functional implications of their EV preparations, with focus on their miRNA contents.

## Material and Methods

Additional details on methodologies are provided in the supplementary material.

### Human BALF samples

Collection and processing of BALF samples from healthy volunteers (HVs) and from ARDS patients was done as previously described [22–24]. In brief, HVs underwent standard fiberoptic bronchoscopy of the right middle lobe at Dartmouth-Hitchcock Medical Center (Lebanon, NH) between January and July 2018 under appropriate institutional IRB protocols. Exclusion criteria for HVs were: history of cardiopulmonary disease, regular smoking or vaping, and use of immunomodulatory medications. BALF samples from ARDS patients without sepsis were collected prospectively as part of an unrelated clinical investigation conducted by the National Heart Lung Blood Institute (NHLBI) ARDSNET (ClinicalTrials.gov NCT0011216) [27]. For the HV lavages, 20 ml sterile saline was utilized, and samples were centrifuged, and supernatants stored at −70°C. For the ARDS patient lavages, a standard 40 ml mini-BALF with sterile saline was utilized in intubated ARDS patients and BALF samples similarly centrifuged and stored. All participants provided written informed consent.

### In vitro exposure of hMSCs to BALF

hMSCs from three healthy donors were obtained from the NHLBI’s Production Assistance for Cellular Therapies (PACT) program (University of Minnesota). The hMSCs have been previously characterized according to criteria from the International Society for Cell and Gene Therapy [2,7,22]. hMSCs were utilized at passages 3–5 and were the same as those used in a recent trial of hMSC administration in nonCOVID ARDS patients (NCT01775774, [2,7]) and in our previous investigations of the effects of BALF on hMSC gene and protein expression [22–24]. hMSCs were seeded into 6-well plates (Corning, 2 × 10^5^ cells/well, 2 wells/BALF sample or control) in cell culture conditions outlined above and incubated overnight. The next day, cells were washed twice with PBS and synchronized for 24 hours in serum-free medium. After synchronization, the serum-free medium was replaced with 1 ml of serum-free medium containing either individual ARDS (N=16) or individual HV (N=16) BALF samples at a 20% (v/v) concentration [22–24]. Control hMSCs were exposed to serum-free medium only (N=16). After 5 hours incubation at 37°C in a standard tissue culture incubator, cell culture medium was removed, cells were washed once with PBS, and 2 ml serum-free medium added per well. After 48 hours incubation (37°C), the conditioned medium was collected, passed through a 0.8 *μ*m syringe filter, and processed for EV preparation, NTA, and miRNA assessments, as described below.

### Extracellular vesicles (EVs) preparation and miRNA isolation

EV preparations obtained from conditioned medium collected from hMSCs exposed to serum-free medium or to individual HV or ARDS BALF samples or directly from pure BALF samples obtained from HVs (N=4) and ARDS patients (N=4) were prepared using the exoRNeasy Serum/Plasma Maxi/Maxi Midi Kit (Qiagen, Germantown, MD, USA), according to manufacturer’s instructions. Sample preparations were stored at −20°C until miRNA sequencing was performed. For nanoparticle tracking analysis (NTA) and imaging flow cytometry analysis, EV preparations were enriched using ExoQuick-TC (cat # EXOTC50A; System Biosciences, Palo Alto, CA) according to the manufacturer’s protocol. Detailed protocols on NTA and imaging flow cytometry analyses are provided in the supplement information section.

### miRNA sequencing and Differential Expression Analysis

RNA was isolated from each EV preparation as described above and only samples that passed quality control with an A260/A280 above 1.80 were used for RNA sequencing (control=16; ARDS=12; HV=14). A total of 35 ul (35 ng/*μ*l) of eRNA (EV preparation-derived RNA) was used for miRNA sequencing performed using the HTG EdgeSeq miRNA Whole Transcriptome Assay (miRNA WTA, as per manufacturer’s instructions as published). The miRNA library was sequenced on a NextSeq (Illumina, Inc., San Diego, CA) using a V3 150-cycle kit with two index reads. PhiX (Roche, Mississauga, ON, CAN). Data were returned from the sequencer in the form of demultiplexed FASTQ files, with one file per original well of the assay. The HTG EdgeSeq Parser (v. 5.0.535.3181, HTG Molecular, Tucson, AZ, USA) was used to align the FASTQ files to the probe list to collate the data. Data has been deposited in Gene Expression Omnibus (GEO, GSE282919). Raw read counts for the 42 samples were imported into R to perform differential expression analyses with the R package DESeq2 [28]. Variance stabilization transformation was used to prepare data for analyses. Significance analyses of microarrays (SAM) was run using one class analysis approach to identify miRNAs over-represented in EVs derived from control hMSCs based on the normalized data using variance stabilization transformation [29]. The delta value was set to 7 (the best delta parameter value selected by the software with the lowest False Discovery Rate (FDR]) using 1000 permutations, FDR cut-off was 0.001 (%).

Target prediction and functional analyses were conducted using miRNet (https://www.mirnet.ca) [30]. Enriched Reactome pathways were selected by hypergeometric tests of miRNAs having FDR ≥ 0.05. Putative relationships were obtained using DIANA-TarBase v8 (collection of experimentally supported miRN–gene interactions) [31].

### Supervised classification analysis for ARDS, HV, and control hMSC-EV preparation miRNAs

Sparse Partial Least Squares (PLS) discriminant analysis (sPLS-DA) (R package “mixOmics”) was performed to identify and narrow the list of miRNAs regulated by ARDS exposure, using a different analysis approach, and narrow the miRNAs to those that were not only differentially expressed but also able to discriminate between treatment groups. The area under the receiver operating characteristic (AUROC) curve for all-vs-one comparisons for PLS-DA are based on predicted maximum distances averaged over all cross-validations and complement the analysis rather than evaluate model performance.

### In silico target predictions for transcripts, genes, and pathways

IntaRNA [32] was used to predict the most likely targets of the top 14 miRNAs selected to be different between the various treatments to extend miRNet Tarbase predictions considering 10 of the newly identified miRNAs were found not to have experimentally proven targets. Interactions between miRNA and our reference transcriptome (https://bio.tools/intarna) with energy scores in the bottom quartile, *i.e.*, an energy score less than −20.7, were categorized as strong predicted targeting to focus on predicted targeting interactions most likely to degrade message or interfere with translation. To estimate the overall targeting of a gene by the 14 miRNAs collectively, we assumed a simple additive model and counted the number of times each transcript of a gene was strongly targeted by any of the 14 miRNAs. Interference at the pathway level was estimated as the total number of strong targeting interactions for all the KEGG genes on a given pathway identified by the KEGGREST R package. Finally, the most strongly targeted pathways relevant to ARDS were visualized using the R package pathview (https://pathview.uncc.edu/home).

### Human bronchial epithelial cells

Primary wild-type human airway epithelial cells (HBEC) were obtained from Dr. Scott Randell (University of North Carolina, Chapel Hill, NC) and cultured as previously described [33]. The Dartmouth Committee for the Protection of Human Subjects determined that the use of HBEC in this study is not considered human subject research as cells are taken from discarded tissue and contain no patient identifiers. Briefly, HBEC from passages 4 and 5 were grown in standard cell culture conditions (37 °C, 5% CO_2_) in BronchiaLife basal medium (Lifeline Cell Technology, Frederick, MD) supplemented with the BronchiaLife B/T LifeFactors Kit (Lifeline) as well as 10,000 U/mL penicillin and 10,000 *μ*g/mL streptomycin (Sigma-Aldrich, St. Louis, MO). Results were independent of passage number. The absence of mycoplasma was verified by routine analysis. HBEC with a viability of 96%–98% were seeded at 2 × 10^6^ cells per T175 cell culture flask and grown to confluence while changing the growth medium every 2–3 days. For measurements of CFTR Cl^−^ secretion and analysis of cytokine and chemokine secretion, HBEC were seeded at 500,000 onto 12-mm Snapwell permeable supports (Corning, Corning, NY) coated with 50 *μ*g/ml Collagen type IV (Sigma-Aldrich, St. Louis, MO) and grown in an air-liquid interface media (ALI) at 37°C for 3–4 weeks to establish polarized monolayers, as described previously [34,35].

### Measurements of CFTR Cl^−^ Currents

To examine the ability of hMSC-EV preparations to influence CFTR Cl^−^ secretion by HBEC, cells were exposed to EVs (2 × 10 [7] particles) or an equal volume of process control (PC: media not exposed to HBEC and run through the EV preparation isolation approach). EV preparations were added to the apical side of HBEC for 6 hours and then removed. CFTR Cl^−^ secretion was measured in the same HBEC monolayers (at the 6-hour and 24-hour timepoints) that were used to assess cytokine secretion, after the medium was removed for cytokine analysis. As described in detail elsewhere [34,36], cells on Snapwell filters were mounted in Ussing chambers whereupon the transepithelial voltage was clamped to 0 mV, and the short circuit current (*I*_sc_) was measured as described previously [34,36]. Subsequently, amiloride (50 *μ*M) was added to the apical solution to inhibit sodium reabsorption. Thereafter, CFTR Cl^−^ secretion was stimulated with forskolin (10 *μ*M; Sigma-Aldrich), followed by thiazolidinone (CFTR_inh_-172, 20 *μ*M; Millipore, Billerica, MA) an inhibitor of CFTR Cl^−^ secretion. Data are expressed as the CFTR_inh_-172 inhibited I*sc*, which is presented as *μ*A/cm^2^. Data were collected and analyzed using the Data Acquisition Software Acquire and Analyze (Physiologic Instruments, San Diego, CA). Although there was some variability in the response of primary HBEC to EV preparations, cells from both donors responded in the same qualitative way.

### Statistical Analyses

To determine differential expression of miRNAs found in BALF-derived EV preparations and hMSC- EV preparations, unpaired T-tests with Welch correction (does not assume equal standard deviations) were performed for those that passed a test for normality (Kolmogorov–Smirnov). For those that were not normally distributed, two-tailed Mann-Whitney tests were performed on a miRNA-by-miRNA basis (*P* = 0.05). Statistical analyses were performed using GraphPad Prism software. The Mann-Whitney test was used to assess differences between groups (comparing top 20 differentially expressed miRNAs detected in EV preparations with those present in BALF from patients). *P* values ≤ 0.05 were considered as significant, except in the case of RNA sequencing data analyzed in DESeq2, where a multiple hypothesis corrected FDR less than 0.05 was significant. Spearman correlations were calculated in base R, using the t distribution to calculate *P* values in those cases that included ties in rank. Significant differences in current were determined using linear models of current as a function of donor and type of EV preparations exposure using PC as the reference condition.

## Results

### BALF-exposure does not significantly affect particle number or size distribution, but decreases number of CD63^+^ objects

**Fig. 1.**
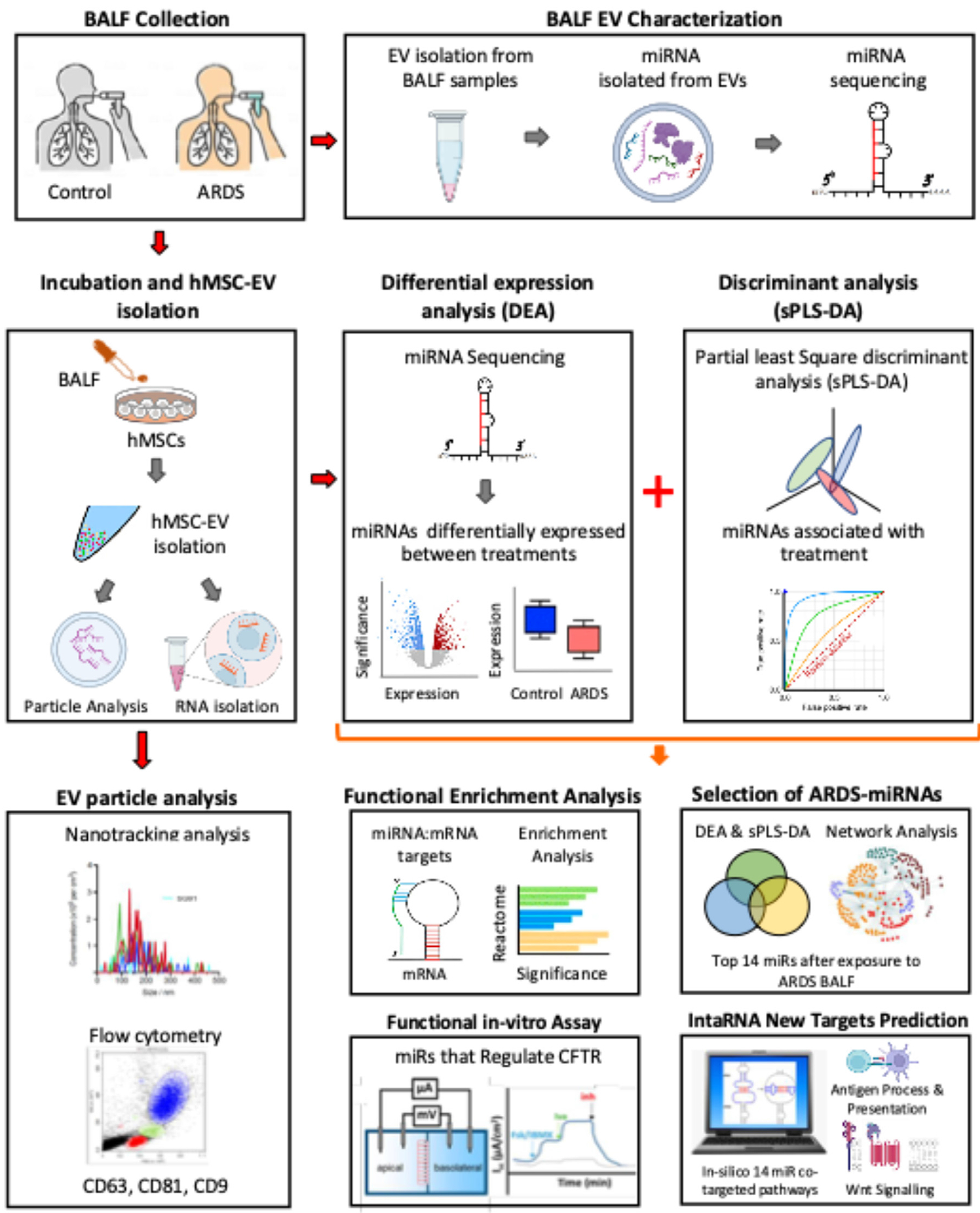
Schematic of experimental design. Human bone marrow-derived mesenchymal stromal cells (hMSCs) were exposed to bronchoalveolar lavage fluid (BALF) from healthy volunteers (HV) donors or acute respiratory distress syndrome (ARDS) patients for 5 hrs. Following removal of the BALF and washing, the hMSCs were then incubated for 48 hours in serum free medium. Extracellular vesicles (EVs) contained in conditioned medium, as well as from the BALF samples themselves, were then prepared and characterized for particle number, size distribution, and expression of characteristic cell surface markers. MiRNAs from purified EVs were sequenced and analyzed. MiRNAs which met statistical cutoffs for differential expression were further analyzed to identify known gene targets involved in inflammation, immunity and response to injury. Independent Partial Least Square Discriminant Analysis (sPLS-DA) was used to identify EV-miRNAs that classified samples into specific treatment groups. Overlap Analysis was used to overlay results from DEA and sPLS-DA and narrow the list of miRNAs selected to identify fourteen EV-miRNAs that were preferentially enriched in EVs derived from hMSCs exposed to ARDS-BALF. Network analysis established 4 of these EV-miRNAs form a regulatory “hub” with their known empirically defined gene targets (TargetBase) enriched for genes involved in cellular stress and Wnt signaling. The other ten EV-miRNAs were relatively novel and had few to no known targets. IntaRNA was used to identify novel in silico predicted targets for the ten novel miRNAs. Using this approach, we determined the collective action of the fourteen ARDS-responsive EV-miRNAs to co-target genes in the same pathways know to play critical roles in acute responses to immune challenges, inflammation and injury. The top pathways (after correcting for multiple comparisons) predicted to be impacted by these miRNAs are antigen processing and presentation and Wnt signaling. Ussing chamber studies measuring CFTR-dependent chlorideflux were used to assess effects of and correlations with EVs containing miRNAs known to regulate CFTR expression. (Color version of figure is available online.)

An overall schematic of the study is presented in Figure 1 summarizing the experimental protocols and analytical approaches utilized. The source and use of individual human BALF samples in the current experiments are presented in Supplementary Table 1. HIPAA limitations precluded sharing of information regarding underlying etiology of ARDS, patient demographics, or the clinical course.

As per suggestions published by the International Society of Extracellular Vesicles (ISEV), global quantification of the number of EV preparations obtained as well as expression of characteristic cell surface markers CD9, CD63, and CD81 following hMSC exposure to the different individual BALF samples was determined [37]. Using nanoparticle tracking analyses (NTA) to assess the number and size of detected particles demonstrated that exposure to control (serum-free) medium or to either ARDS or HV BALF resulted in 90% of particles in the hMSC secretome sized between 50 and 200 nm (Supplementary Figure 1A). The number of particles collected was not significantly different after HV or ARDS BALF exposure, but the number of particles collected in these two conditions were each significantly greater than those collected from control (serum-free medium) exposure. Image flow cytometry demonstrated no difference in the content of CD81^+^ objects between experimental groups but both ARDS and HV BALF exposure significantly altered the number of CD63^+^ and CD63^+^/CD81^+^ objects compared to control (Supplementary Figure 1B). CD9^+^ objects were not detected in any of the EV preparations (data not shown). In comparison, particles contained in EV preparations directly obtained from the BALF samples themselves encompassed a wider size range with more particles in ARDS BALF samples (Supplementary Figure 1C). There was a significant higher content for CD81^+^ objects in HV BALF, a nonsignificant increase in the CD81^+^ object content in ARDS BALF, and a trend towards higher CD63^+^ objects in both ARDS and HV BALF samples compared to control. In contrast to the hMSC-EV preparations, there were detectable amounts of CD9^+^ objects in both ARDS and HV BALF EV preparations (Supplementary Figure 1D). Gating strategies and individual scattergrams are shown in Supplementary Figure 2. In summary, BALF exposure did not affect hMSC origin particle number or their average size distribution but decreased the number of CD63^+^ objects. The implications of this are currently unknown.

### EV preparations derived from control hMSCs contain miRNAs predicted to down regulate inflammatory pathways

**Fig. 2.**
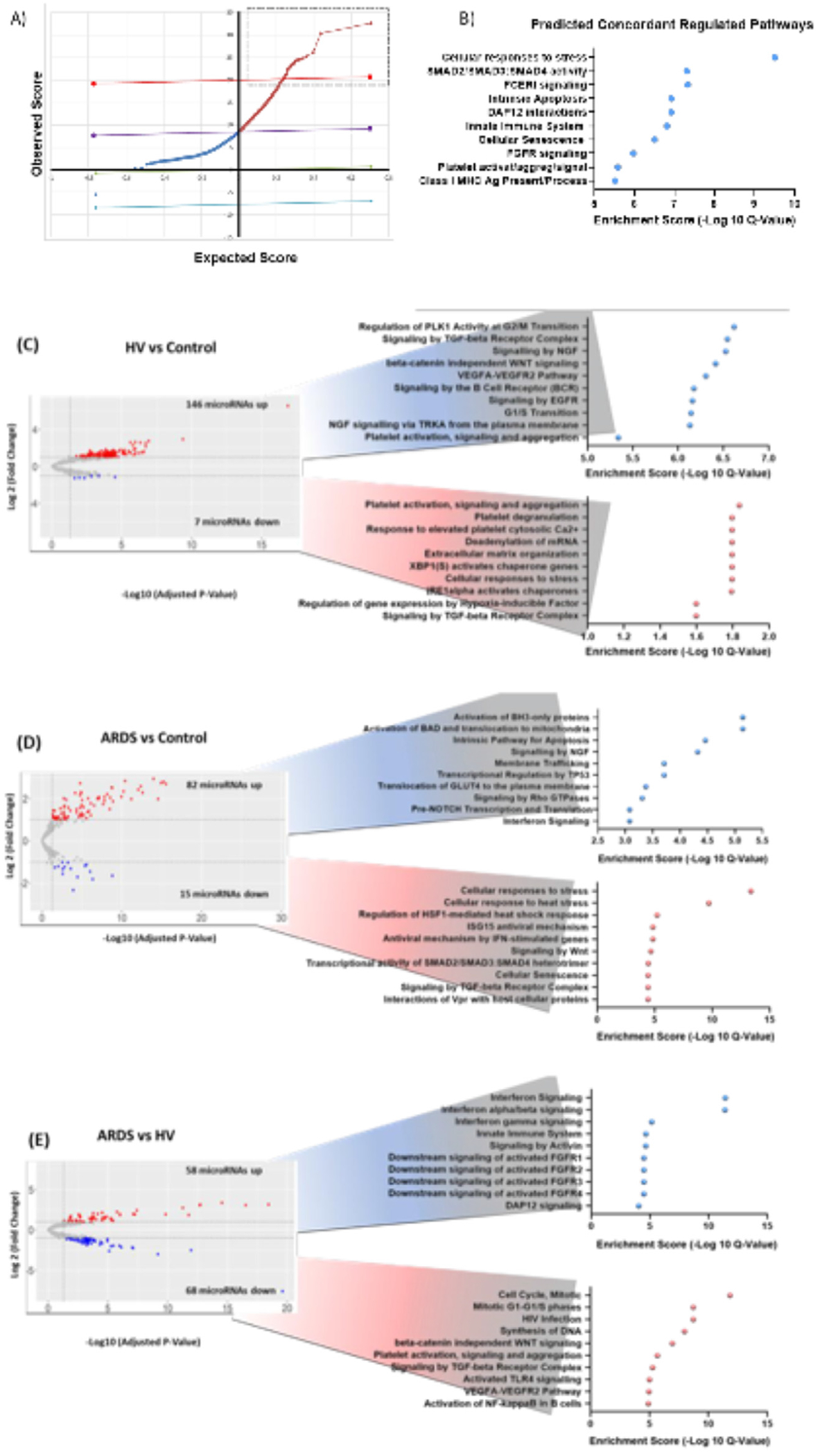
Treatment with HV vs ARDS BALF results in different miRNAs being differentially expressed in hMSC-EVs. (A) Significance Analysis of Microarray (SAM) output plot showing miRNAs that were over-represented in EVs derived from control hMSCs alone. The *δ*-value in SAM was set to 7 (best delta selected by software for lowest False Discovery Rate [FDR]), and the FDR was 0.001 (%). A total of 770 miRNAs were observed to be present above the expected rate compared to the average expression of all miRNAs across replicates (above the upper delta line in purple). MiRNAs between the upper and lower delta line (in blue) were considered not significantly changed compared to the average abundance across all replicates. A total of 48 miRNAs were markedly over-abundant (score ≥ 20 above cut-off red line, in gray square dashed line). Median Number of False Positives =0; Tail strength (%) = 99.4; se (%) = 153.7. TarBase v8 (collection of experimentally supported miRN–gene interactions) was used to identify known targets of these miRNAs and miRNet utilized to predict which pathways would be most affected by the overabundance of these miRNAs (enriched terms filtered by hypergeometric test cut-off = adjusted *P* value ≤ 0.05). (B) The top 10 REACTOME terms are shown. The Log10 q-value (FDR) of the enrichment score is plotted (blue circles). Volcano plots of differentially expressed miRNAs (fold change (FC) in normalize counts >2 of miRNAs vs false discovery rate (FDR) <0.05 expressed as the negative Log_10_ of the FDR) for pairwise comparisons: (C) Control vs healthy volunteer (HV); (D) ARDS vs control, and (E) ARDS vs HV. Total number of miRNAs fulfilling differential expression criteria are stated for each plot. Each dot in the volcano plot represents a miRNAs and are colored by FC (by convention, red FC≥2; blue ≤−2). In the volcano plots, miRNAs that were not changed are shown as gray dots. Dashed lines correspond to FC and FDR cut-offs. TarBase v8. Pathways predicted to be affected by increase or decrease in EV-miRNAs were identified in miRNet. The top 10 enriched pathways, ranked by adjusted P value < 0.05 (expressed as −Log10 of q-value) from hypergeometric test are presented; pathways are circles, colored by the expected direction of change depending on increased or decreased expression of the miRNA in the comparison (by convention blue is decreased and red is increased activity). (Color version of figure is available online.)

Before investigating the effect of BALF on the miRNA content of the EV samples, miRNAs were profiled in EV preparations obtained from control hMSCs (exposed to serum-free medium only, control, N=16). To determine if specific miRNAs were more abundant than expected, we used significant analysis of microarray (SAM) to identify those miRNAs with count number significantly above the average count of all miRNAs across all control biological replicates (score ≥ 20, delta [*δ*] was set at 7 as per default criteria (False Discovery Rate [FDR] = 0.001), Figure 2A). Of the 770 miRNAs present in these EVs, 48 were markedly over-represented – meaning that they were found at a count number significantly above the expected range (expected/observed). This includes miR-138-5p, and miR145-5p, both implicated in CFTR regulation as further discussed below. A list of all miRNAs present in control hMSC-EV preparations ranked by their overrepresentation score is presented in Supplementary Table 2. Enrichment analysis for these 48 miRNAs using miRNet based target prediction (DIANA miRTarbase v8.0,[31]) demonstrated that highly expressed miRNAs are predicted to regulate genes involved in cellular response to stress, SMAD activity, and FC receptor signaling among other pathways (Figure 2B and Supplementary Table 3).

### miRNAs content in hMSC-EV preparations are altered by BALF exposure

Next, we sequenced and compared miRNAs obtained from EV preparations derived from hMSCs exposed to BALF from ARDS patients (ARDS, N=12), or healthy volunteers (HV, N=14) to controls (N=16). Principal component analysis (PCA) using normalized unfiltered miRNA counts, revealed clustering of samples by treatment assignment (Supplementary Figure 3A).

To further determine the effect of BALF exposures on EV preparation-miRNA content, three pairwise comparisons were performed: HV versus control (differentially expressed (DE) = 153 miRNAs), ARDS versus control (DE = 97 miRNAs), and ARDS versus HV (DE = 126 miRNAs; results in Supplementary Table 4). miRNAs were deemed to be differentially expressed if they met statistical cut-offs of more than a two-fold change in expression (Log2 fold change [FC]) and an FDR ≤ 0.05. Volcano plots and top 10 pathways predicted to be concordantly regulated for each comparison are shown in Figures 2C–E and Supplementary Tables 5–7. Compared to controls, EV associated miRNAs from hMSCs exposed to HV BALF are predicted to downregulate genes involved in cell cycle, TGF*β*, VEGF, and EGFR signaling. In contrast, miRNAs that were less abundant are predicted to be involved in extracellular matrix organization and activation of hypoxia inducible factor (Figure 2C). Compared to control, EV preparations from hMSCs exposed to ARDS BALF demonstrated an overabundance of miRNAs predicted to inhibit genes involved in cellular response to stress and interferon signaling, as well as BH3-only proteins (*e.g.* Bax and Bad, selective trigger of canonical mitochondrial apoptosis in response to developmental cues), and stress-signals like DNA damage (Figure 2D). In terms of differences between HV and ARDS BALF-exposed hMSC-EVs, genes impacted included those involved in cell cycle, DNA synthesis, Toll like receptor 4 (TLR4), and NF-kappa B activation after exposure to ARDS BALF. In contrast, EV preparations from ARDS-exposed hMSCs contain miRNAs that regulate genes involved in interferon, activin (related to TGF-*β*), and FGFR signaling (Figure 2E). In summary, after exposure to ARDS BALF, EV preparations contain an overabundance of miRNAs that are predicted to reduce inflammation.

Accordingly, to determine if EV preparations prepared from hMSCs exposed to the different BALF samples had a functional effect on cytokine secretion of pulmonary cells, primary human bronchial epithelial cells (HBECs) were grown in air liquid interface culture and exposed to EV preparations for 6 hours, whereupon analysis of 48 cytokines in the conditioned media were performed 6-hours or 24-hours after initiating exposures. Exposure of HBECs to control, EV preparations, HV-EV preparations, or ARDS-EV preparations for either 6 or 24 hours had no significant effect on the levels of any of the 48 pro- or anti-inflammatory cytokines and other mediators assessed including IFN-*γ*, IL-6, IL-10, and others (Supplementary Table 8).

### The effect of EV preparations on CFTR Cl^−^ currents is time and EV preparation type dependent

**Fig. 3.**
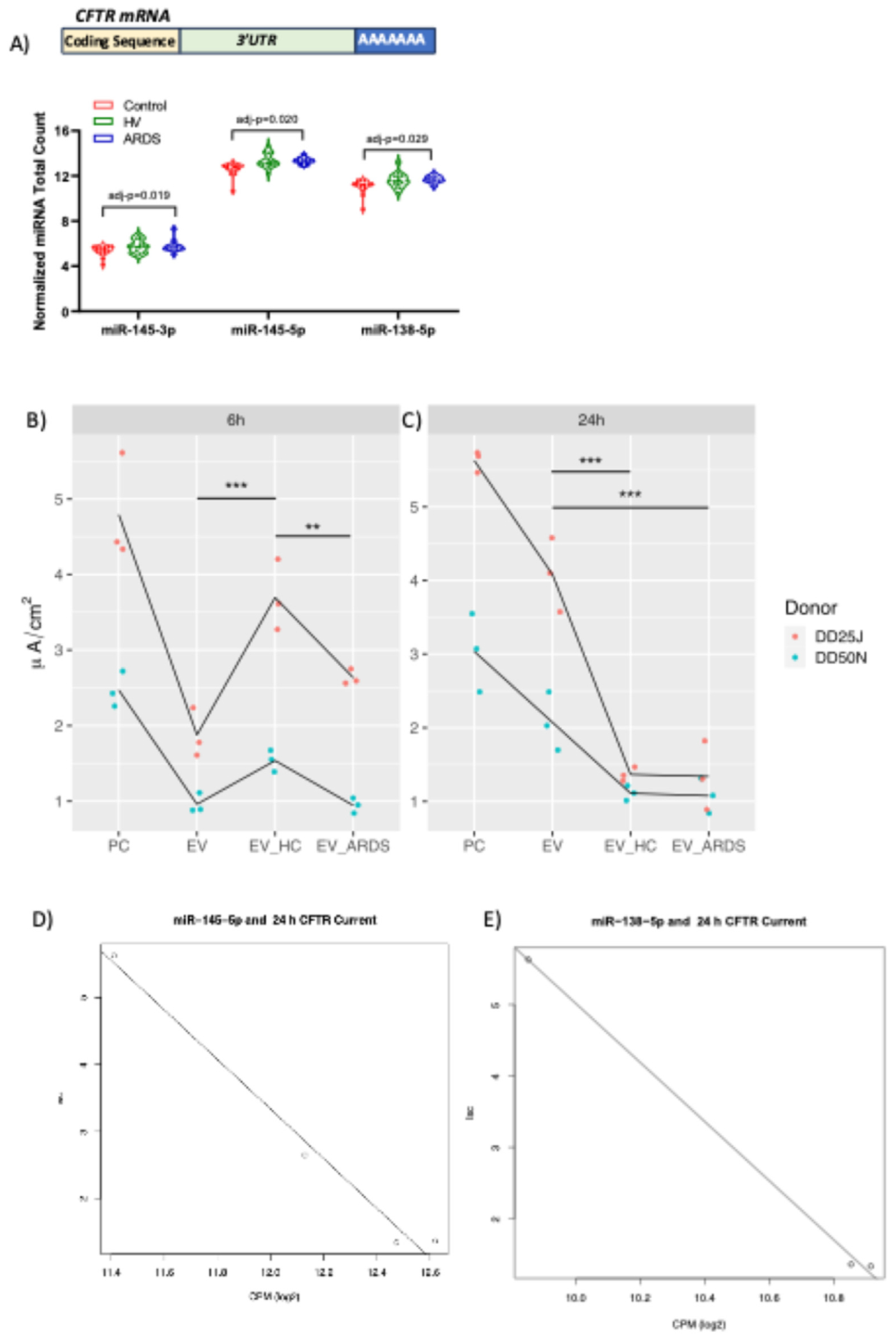
EVs reduced CFTR Cl^−^ secretion by HBEC. (A) edgeR analysis of differential gene expression revealed that only miR-145-3p, miR-145-5p, and miR-138-5p were upregulated in EVs secreted by hMSC exposed to ARDS BALF compared to control and HV EVs (*P* < 0.01). (B) HBEC exposed to process control (PC), EVs secreted by hMSC exposed to vehicle (EV), EVs secreted by hMSC exposed to BALF isolated from healthy controls (EV HC) and EVs secreted by hMSC exposed to BALF isolated from individuals with ARDS (EV ARDS) 6 hours after exposure to EVs. ***P < 0.001, **P < 0.01, N.S., not significantly different. (C), HBEC exposed to PC, EV, EV HC and EV ARDS 24-hours after initiating exposure to EVs. **P < 0.01 and ***P < 0.001 versus PC. Data expressed as the forskolin-stimulated, CFTR-172 inhibited Cl- secretion (*μ*A/cm2). Each data point represents one monolayer of HBEC grown in air liquid interface (ALI) culture. Lines connect HBEC data from the same donor. Linear models were used to compare treatment groups to each other and to take donor differences into account. (D), Correlation curves demonstrating association of miR-138-5p and miR-145-5p with EV affects n CFTR Cl− current. Abbreviation: HBEC, human bronchial epithelial cells. (Color version of figure is available online.)

Chloride ion secretion by lung epithelial cells plays a key role in mucociliary clearance of bacteria and other environmental particles from the lungs [36]. A review of the literature reveals that several miRNAs which regulate CFTR Cl^−^ secretion by airway epithelial cells, including miR-200b, miR-101, miR-145, miR-223, miR-494, miR-509–3p, miR-1246, miR-9, miR-138, miR-384, and miR-600 [38] were present in EV preparations secreted by hMSCs. Analysis of differential gene expression using a gene-wise negative binomial generalized linear mode revealed that only miR-145-3p, miR-145-5p, and miR-138-5p were upregulated in EV preparations secreted by hMSC exposed to ARDS BALF compared to control and HV EV preparations (*P* < 0.01) Figure 3A.

Accordingly, to determine if EV preparations prepared from hMSCs under the different exposure conditions affected CFTR Cl^−^ secretion, monolayers of primary HBECs were exposed to EV preparations and CFTR Cl^−^ currents were measured as described in methods. As shown in Figure 3B–C, CFTR Cl^−^ currents in HBEC were ~ 5 *μ*A/cm^2^ in donor DD25J and ~ 3 *μ*A/cm^2^ in donor DD50N at both 6 and 24 hours after exposure to process control (PC). All EV preparations tested reduced CFTR Cl^−^ currents compared to PC at 6 and 24 hours after initial exposure (*P* < 0.01 compared to PC, based on a linear model of current as a function of donor and type of EV preparations using PC as the reference condition). The effect of EV preparations on CFTR Cl^−^ currents was time and EV preparation type dependent. At 6h, HV-EV preparation currents were larger than EV preparation currents (***P = 0.0002). ARDS-EV preparation currents were larger than HV-EV preparation currents (**P = 0.004). The response of HBEC to EV preparations 24h after initial exposure to EV preparations had a different pattern than at 6 h. The current in HBECs exposed to HV-EV preparation and ARDS-EV preparation were significantly lower than EV preparation (***P < 0.001); however, there was no difference between ARDS-EV preparation and HV-EV preparation CFTR Cl^−^ currents (*P* = 0.94). Thus, the effect of EV preparations on CFTR Cl^−^ currents was time and EV preparation type dependent.

**Fig. 4.**
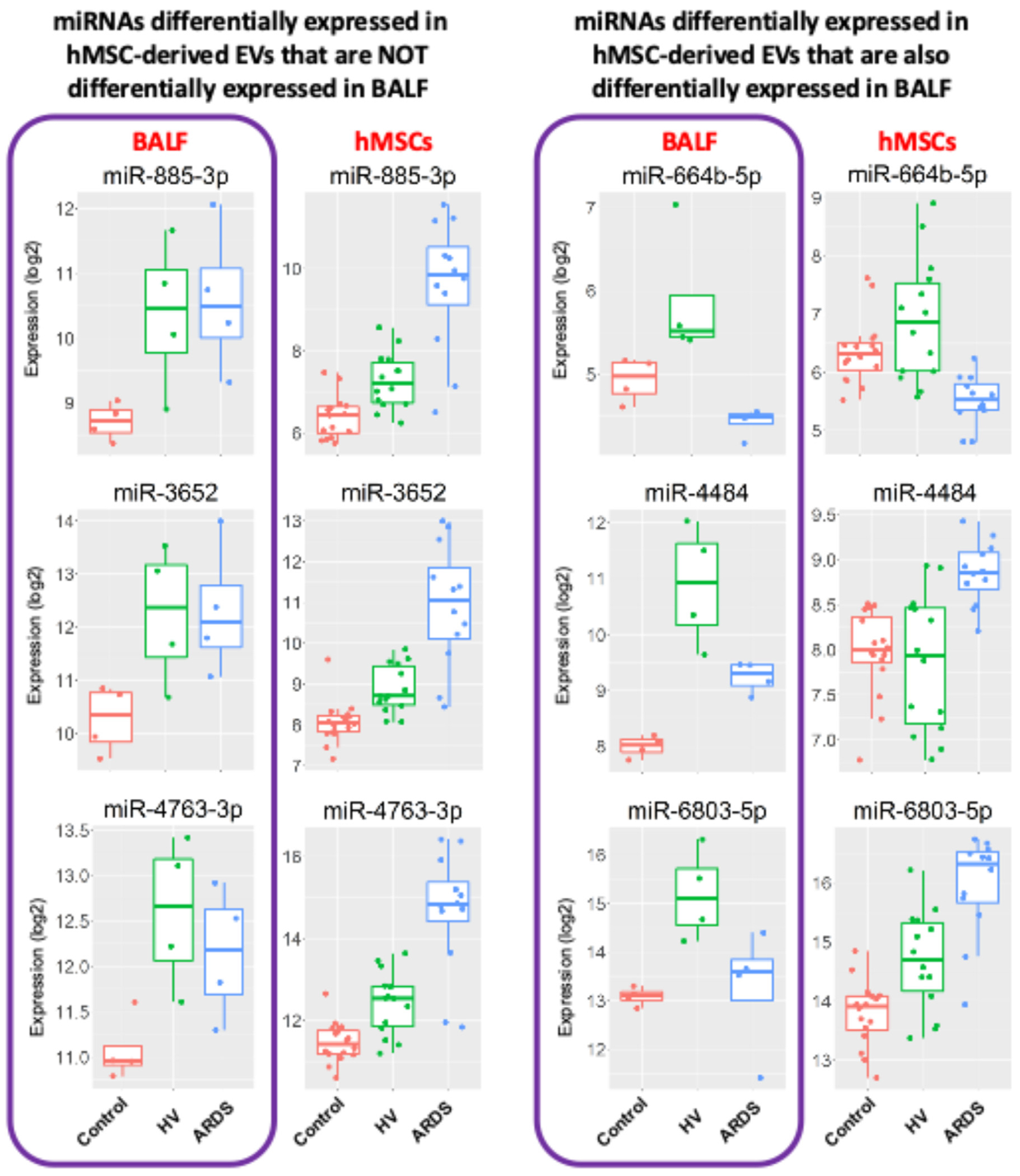
EV-miRNAs are not all the same compared to miRNAs isolated directly from BALF. Box plots showing representative changes in normalized counts (Log2) of miRNAs deemed to be differentially expressed in BALF (purple square) vs those found to be differentially expressed in EVs derived from control hMSCs (red), treated with BALF from HV (green), and ARDS patients (blue). Line in box is the median quartile, squares are upper and lower quartiles and whiskers are maximum and minimum range. BALF, bronchoalveolar lavage fluid samples; control, serum-free medium; HV, healthy volunteer; ARDS, acute respiratory distress syndrome; miR, microRNA; hMSC, human mesenchymal stromal cells; EVs, extracellular vesicles. (Color version of figure is available online.)

To further clarify these results and the possibility that the miRNA content might reflect contamination of BALF EV preparations, miRNAs in EV preparation samples isolated from ARDS BALF (N=4), HV BALF (N=4), and from the serum-free medium (control, N=5) used to incubate the hMSCs were sequenced for miRNA content. Of the top 20 miRNAs differentially recovered in EV preparation samples derived from ARDS BALF-exposed hMSCs, 13 miRNAs were not abundant in EV preparations directly obtained from ARDS BALF compared to HV BALF. Of the 7 miRNAs with different contents in the BALF and differentially present in hMSC-EV preparation samples, 3 had opposite directions of change, strongly suggesting different biologic effects of both ARDS and HV BALF exposures rather than cross contamination from EV preparations found in either the ARDS or HV BALF samples. Representative examples are depicted in Figure 4.

### Supervised classification analysis produces a signature of overlapping discriminatory miRNAs

**Fig. 5.**
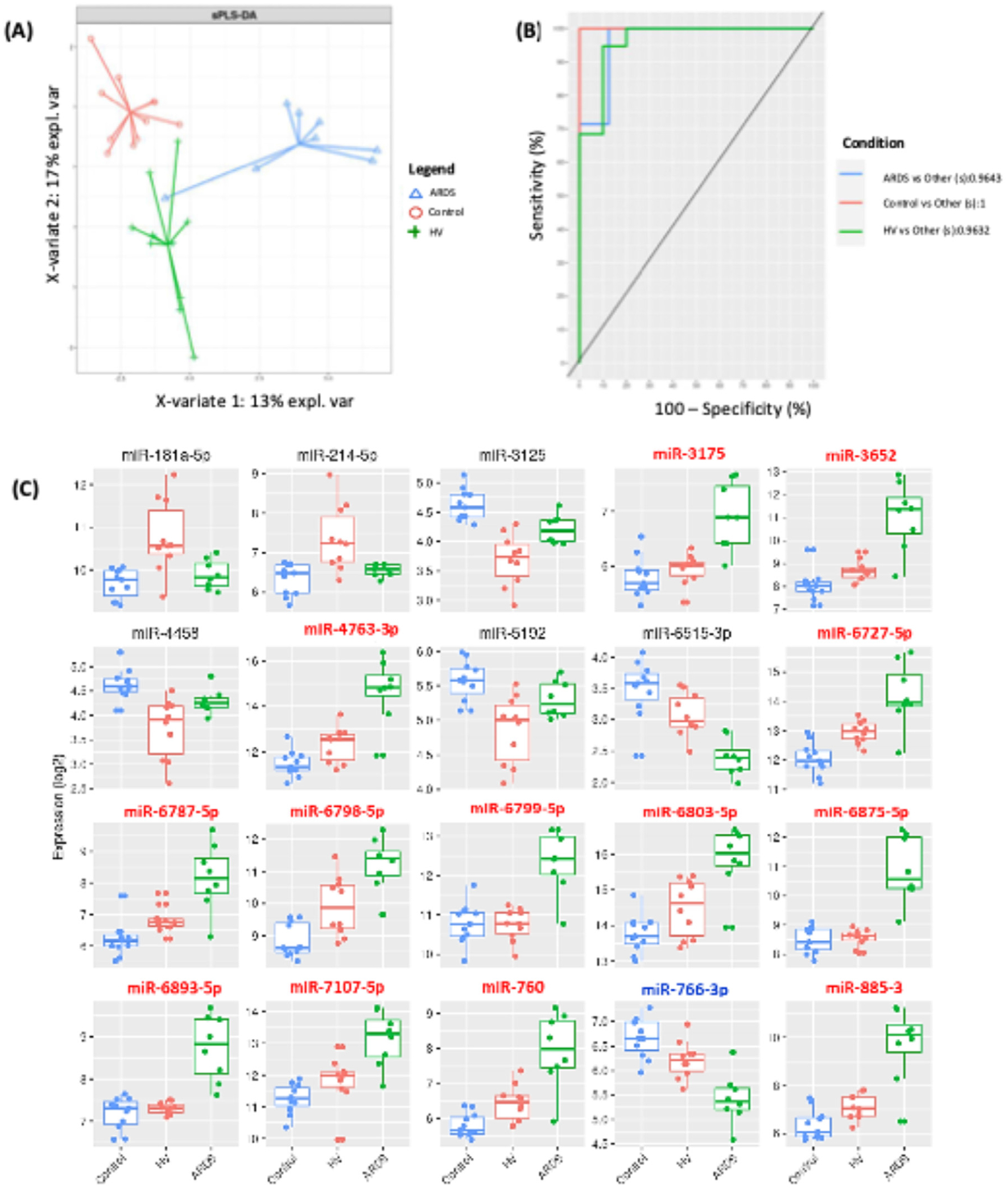
Separate discriminate analysis identified miRNAs able to classify samples into experimental treatment groups. (A) sparse PLS-DA plot where each sample is a point colored by class and lines extend from the group centroid to the individual samples. 20 miRNAs were selected for maximal discrimination between classes. (B) ROC curve with AUC for all-vs-one comparisons averaged over all cross-validations and based on predicted maximum distances. (C) Box plots showing normalized counts of all 20 miRNAs selected as classifiers by sPLS-DA. EV-miRNAs that are also differentially expressed are labelled in red or blue depending on whether the abundance of the miRNA was increased or decreased respectively. Median quartiles are shown as well as upper and lower quartiles; whiskers are maximum and minimum range. ARDS, acute respiratory distress syndrome; Control, serum-free medium; HV, healthy volunteer; miR, microRNA. (Color version of figure is available online.)

We next used an independent statistical strategy to identify those miRNAs able to discriminate between treatment groups. To achieve this, samples were split into training and test sets (70% versus 30%, respectively) and Sparse Partial Least Squares Discriminant Analysis (sPLS-DA) was used to develop the classification model on training data. A 2-component model with 15 and 5 miRNAs selected on the first two components, respectively, was found to have the lowest balanced discriminant error rate (0.0883). A 20-miRNA signature was found to discriminate samples into their appropriate treatment group (ARDS BALF-exposed, HV BALF-exposed, or control) with the first component separating ARDS from the other classes, and the second component providing resolution between HV and controls (Figure 5A). The ability of the 20 miRNAs to accurately classify samples into treatment groups was tested using leave-one-out cross-validation (LOOCV) in the test cohort [34]. The area under the receiver operating characteristic curve for ARDS versus HV + controls, which is based on prediction distances and averaged over all cross-validations, was 0.9643 (Figure 5B). The accuracy of the signature in predicting each class for the test set was 0.92, 0.85, and 0.92 for ARDS, HV, and control groups, respectively. The sensitivity, specificity, and error rate for the test and training sets can be found in Supplementary Table 9.

We then assessed those miRNAs that had been independently selected by both strategies. A total of 14 of the 20 miRNAs identified using sPLS-DA were also differentially expressed between treatment groups (Figure 5C). These miRNAs were in the top quartile of changes in abundance (Supplementary Figure 3) and were discovered using the most statistically robust approach.

Table 1 describes what is known about putative functions of the top 14 miRNAs that may be relevant to lung injury. MiR-766-3p shares direct links to genes involved in cell stress responses, whilst miR-885-3p, miR-3175, and miR-760 each contribute to predicted regulation of genes involved in Wnt signaling (Supplementary Figure 5). MiR-885-3p has direct interaction with genes involved in pro-inflammatory cytokine regulation, apoptosis, chemoresistance, proliferation, and metastasis. MiR-766-3p regulates genes that inhibit inflammation by acting through NF-kB signaling. MiR-664b-3p, miR-4644, miR-6803-5p, miR-6869-5p, miR-3940-5p, and miR-766-3p are implicated in the regulation of proliferation. MiR-3175 promotes epithelial-mesenchymal transition by targeting Smad7. MiR-760 is considered as a tumor suppressor as it negatively regulates oncogenic proteins and decreased proliferation, cell cycle progression, migration, and differentiation. Ten of the 14 miRNAs identified had no known targets (Tarbase v8.0), 3 of which have not as yet been referenced in Genome Ontology (Supplementary Table 10).

In summary, two orthogonal approaches were utilized to identify the miRNAs differentially expressed in hMSC-EV preparations exposed to control, HV, or ARDS BALF. The results indicate that there were 14 miRNAs that are differentially expressed in EV preparations from hMSCs exposed to ARDS BALF that are predicted to reduce lung injury.

### Exposure of hMSC to ARDS BALF results in the overrepresentation of 14 miRNAs that are predicted to collectively regulate antigen presentation and processing and Wnt Signaling

To better understand the biological role of both target-specific and miRNAs that do not have known targets, we used IntaRNA to predict transcripts, genes and pathways targeted by the 14 miRNAs identified above [32]. The IntaRNA algorithm predicts the ability of short RNA to bind to a sequence in a target transcriptome based on sequence complementarity between the short RNA and other factors resulting in an energy score of the predicted interaction. A more negative energy score signifies a stronger interaction and a higher probability of message degradation or interference with protein translation, and those miRNAs with energy scores in the bottom quartile can be characterized as relatively strong interactions most likely to have biological effects. We therefore counted the number of strong interactions between the 14 miRNAs and the transcriptome, summarized strong interactions by gene, and finally calculated the total number of strong targeting interactions for all genes on a given KEGG pathway. The 20 most highly targeted pathways are shown in Table 2.

**Fig. 6.**
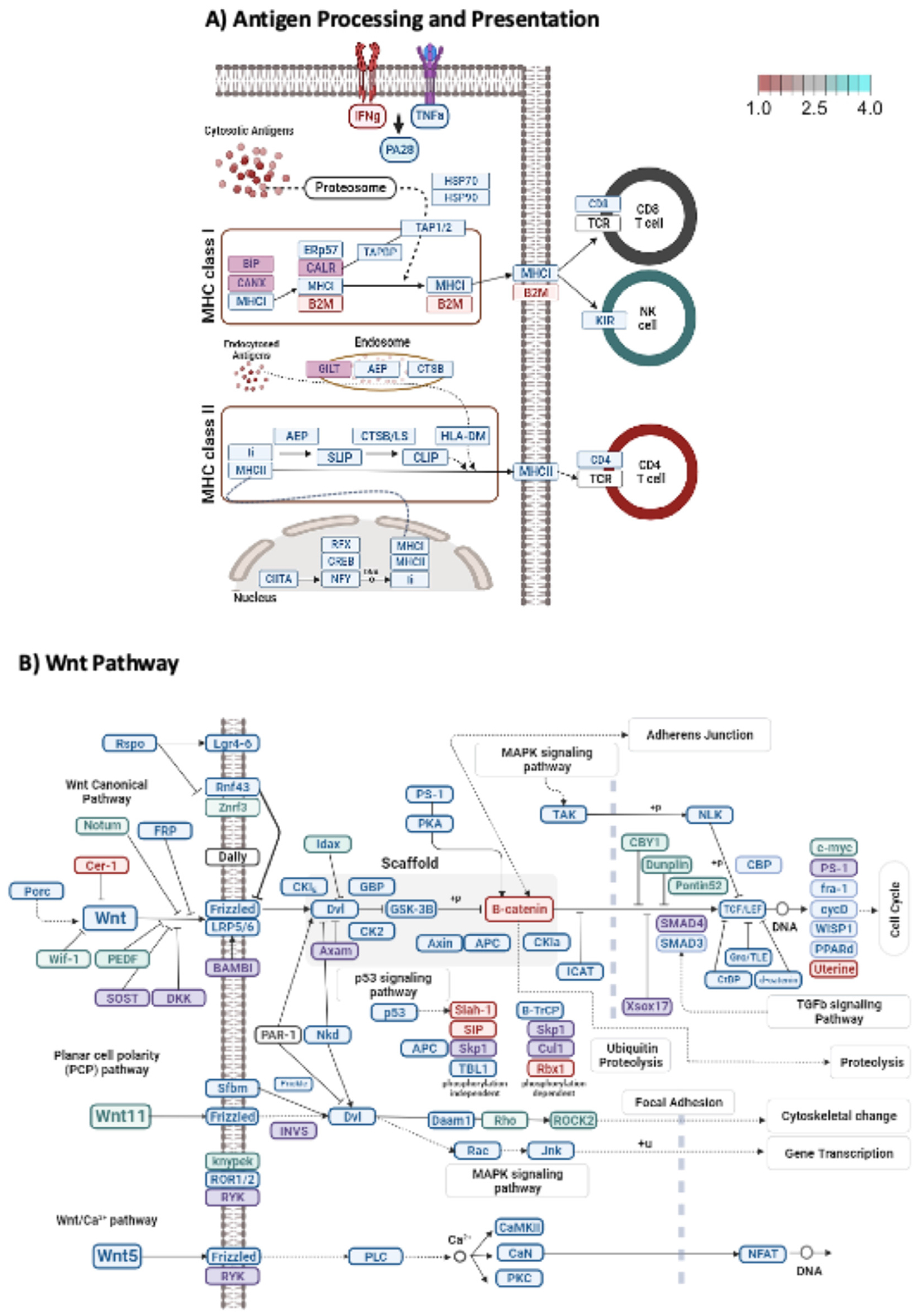
Pathways. Top 14 ARDS-BALF-sensitive EV-miRNAs are predicted to co-target genes in Antigen Processing & Presentation and Wnt Signaling pathways. IntaRNA was used to identify novel genes, not known to be targets of the 14 ARDS BALF-sensitive EV-miRNAs and to estimate the overall targeting of a gene by the 14 miRNAs collectively. An energy score for the miRNA-mRNA interaction less than −20.7 was used for unbiased miRNA-target selection. A simple additive model was assumed and counted the number of times each transcript of a genes was strongly targeted by any of the 14 miRNAs. Interference at the pathway level was estimated as the total number of strong targeting interactions for all the genes on a given pathway. The most strongly targeted pathways relevant to ARDS were visualized using the R package pathview and pathways were drawn using Bio-Render. Selected genes in specific pathways are predicted to be targets of multiple miRNAs. The top two pathways predicted to be targeted (A) Antigen Processing and Presentation (B) and Wnt signaling. Targets are colored by number of hits and the expected direction of change for each gene targeted in the pathway. By convention predicted increased expression levels are red, and decreased expression levels are blue (reference bar is shown in upper right corner). (Color version of figure is available online.)

To further explore pathways of special interest in ARDS (shown in bold in Table 2) we prepared visualizations of the relative targeting of each gene on each respective path. As shown in Figure 6, IntaRNA predicts that many genes in each of these paths were themselves highly targeted. The genes most highly targeted by the 14 miRNAs are in the fourth quartile of strong target events (**blue**) and the least targeted genes are in the first quartile (**red**), reflecting the expected direction of change. In keeping with previous work from our group [26], and consistent with our analysis above, we found that the top pathways (after correction for the size of the pathways) predicted to be regulated by the 14 miRNAs differentially present in EV preparations derived from hMSCs exposed to ARDS BALF are antigen processing and presentation, Wnt signaling, cytokine-receptor interactions, cell adhesion molecules, MAPK and calcium signaling, natural killer cell mediated toxicity, and focal adhesion pathways.

## Discussion

There remains a fundamental lack of knowledge as to the fate and actions of exogenously administered hMSCs and their EV preparation products in clinical lung disease inflammatory environments. We have previously demonstrated that the ARDS inflammatory environment, utilizing clinical BALF as a surrogate, has profound influence on hMSC gene and protein expression, including regulation of HLA-related gene and protein expression [22–24]. EV preparations are increasingly recognized as mediators of the anti-inflammatory effects and other biological activities of their parent hMSCs [39]. This has been found in preclinical models of acute lung injury and other lung diseases, where EV preparations are as and sometimes more effective than the parent MSCs themselves [5,40]. These laboratory findings have spurred on initial clinical investigations of hMSC-EV preparations in patients with ARDS, bronchopulmonary dysplasia, and other lung diseases [8,41]. However, the mechanisms by which hMSC-EVs influence the inflammatory lung environment are still being elucidated.

We now demonstrate that the ARDS inflammatory environment also influences hMSC-EV preparation miRNA content. Using two different bioinformatic strategies, DEA and sPLS-DA, 14 miRNAs were found to be significantly enriched in EVs derived from hMSCs exposed to ARDS BALF compared to HV BALF and control. 13 of these were increased, while one was decreased in abundance relative to the other treatment groups. Network analysis of the 4 miRNAs with known gene targets (miR-760, miR-3175, miR-885-3p, and miR-766-3p), demonstrated a regulatory “hub” modulating the expression of genes involved in cellular response to stress and to Wnt signaling. This is notable as the Wnt/*β*-catenin pathway has been implicated in the induction, promotion, and abnormal repair of acute lung injury in many preclinical models [42,43]. However, the other 10 miRNAs over-expressed in EV preparations from ARDS-treated hMSCs are not well studied, with few to no documented targets.

*In-silico* target prediction for all 14 miRNAs, including the 10 novel miRNAs, identify co-targeting of various pathways fundamental to innate immune responses, such as antigen processing and presentation. For example, regulation of MHC class genes by miRNAs has been well documented as a strategy to control innate immune and inflammatory activation [44,45]. We have previously shown that HLA-related genes are significantly decreased in hMSCs exposed to ARDS BALF [24], but not much is known about whether hMSC-EV preparations regulate HLA class proteins in recipient cells. The role of hMSC-EV miRNAs in innate immune regulation is further reinforced by our data demonstrating predicted action of these miRNAs in the downregulation of genes involved in cytokine-cytokine receptor interaction, cell adhesion, MAPK and calcium signaling, and cytotoxicity. Thus, additional investigations will be required to determine the biological effects of these newly identified EV preparation miRNAs and their relevance to acute lung injury. We also assessed effects on a single cell type to date, and it may well be those effects on inflammatory mediator production in other cell types, for example endothelial cells or macrophages, may be more relevant.

Interestingly, impaired CFTR function have been observed in infectious and inflammatory diseases, and importantly, Erfinanda *et al.* found that a rapid decreased of CFTR function were linked to pneumonia-induced ARDS after *Streptococcus pneumoniae* infection [46,47]. These results parallel data from Honrubia *et al.*, in which they demonstrated that SARS-CoV-2 infection in mice resulted in decreased CFTR expression, a loss of function that were elegantly reversed by treatment of the CFTR potentiator Ivacaftor [48]. Our data support this link between ARDS and impaired CFTR function. In the current study we found that EV preparation samples obtained from ARDS BALF-exposed hMSCs significantly reduced CFTR Cl^−^ secretion by primary HBECs, an effect predicted to reduce mucociliary clearance of bacteria and other environmental particles [49]. Based on the observation that miR-435-5p, miR-345-3p, and miR-138-5p are present in all EV preparation samples and have been shown to inhibit CFTR Cl^−^ secretion [50,51] we suggest that miR-435-5p, miR-345-3p, and miR-138-5p may be at least in part responsible for the EV preparation-mediated decrease in CFTR Cl^−^ secretion. Additional studies, beyond the scope of the present study, using miRNA antagomirs, are required to prove that miR-435-5p, miR-345-3p, or miR-138-5p alone may be responsible for the EV preparation-mediated decrease in CFTR Cl^−^ secretion. At present, the significance of these findings remains unknown and needs to be further investigated.

This study has several strengths. First, the bone marrow-derived hMSCs utilized are clinically relevant, having also been utilized in a recent trial of systemic hMSC administration in ARDS patients [2,7]. Second, the BALF-exposed EV preparation samples were assessed individually rather than as pooled samples. As such, the current findings are robust and reproducible across multiple individual clinical ARDS or HV BALF samples. Nonetheless, we acknowledge several caveats including the different techniques utilized to collect and store the ARDS versus HV BALF samples, including different volumes of fluids used for the lavages, and that the ARDS population excluded those with sepsis as the underlying etiology [27]. Furthermore, given HIPAA limitations, no clinical data on the underlying etiology of the ARDS patients are available except that for exclusion of patients with sepsis/septic shock, or of their clinical course [27]. Similarly, limited clinical information is available for the HV samples. One additional caveat to this study is that the BALF samples utilized were cell-free supernatants. As such, although these caveats are unlikely to bring the validity of the current observations into question, given the strength of the association between predicted target genes and clinically relevant KEGG pathways (antigen-presentation, Wnt signaling, etc.), further prospective studies will attempt to better control for these variables.

Another limitation with the study reflects ongoing controversies concerning how to best prepare hMSC-EV preparations, whether all particles detected represent EVs, and whether miRNAs detected are associated with the EVs. In the current study, the EV sample preparations assessed for miRNA content were prepared using the exoR-Neasy Serum/Plasma Maxi/Maxi Midi Kit while EVs prepared for NTA and imaging flow cytometry evaluations were prepared using the System Biosciences ExoQuick-TC kit given background contamination from particles other than EVs with the exoEasy Kit. While the latter kit can more cleanly extract EV particles, some data suggests that these might not include the exosomal EV fraction [52]. In addition, the Qiagen kit utilizes silica membranes with nucleic acid affinities. Thus, the EV preparation they enrich might be decorated with nucleic acids, including miRNAs. Therefore, while good for preparation of EV associated miRNAs, these might not be the ones being involved in intercellular communication processes. Ongoing studies will help clarify these issues and will use the most recent MISEV guidelines [53]. Moreover, there is a risk that the prediction tools and pathway analysis used is based on imperfect algorithms and publication bias, therefore additional functional studies such as antagomir studies and functional assays on a range of cells including epithelial cells, endothelial cells, macrophages, and other immune effector cells are needed to verify the findings.

A further issue is clarifying whether the miRNAs identified are directly associated with defined EV particles and with which classes of EV particles. There is growing appreciation that EVs cannot be cleanly isolated, particularly with the techniques utilized for these studies. Other particles such as lipoproteins and proteins are frequently co-isolated, and it is not clear yet whether identified miRNAs are indeed contained in obtained EV samples as EV cargo molecules or as byproducts of the isolation technique [26]. Traditionally utilized approaches such as NTA do not discriminate this. Characterization of EV particles by imaging flow cytometry (IFCM) provides information on EV subtype, as suggested by recent ISEV guidelines. However, this also does not clarify functional miRNA association with any given EV population. Nonetheless, the current findings suggest that EV preparations produced by hMSCs exposed to either ARDS or HV BALF have unique miRNA profiles. Further, hMSC-EVs may not always express CD9 [54] and different EV populations were found in ARDS versus HV BALF itself. We did not assess EV CD14 expression as was recently done in sepsis-associated ARDS BALF samples [55], however, the BALF samples utilized in the current study excluded patients with sepsis [27]. The implications of this are not yet understood, but further assessments of BALF EV preparation content may shed light on both disease pathogenesis as well as new potential therapeutics. The responsible EV components are still being elucidated and in particular the role of miRNAs themselves in mediating EV actions is controversial [25,26,56]. Nonetheless, despite these limitations, the current studies provide further evidence of the plasticity of hMSCs and their EVs and. Importantly, they offer a number of mechanistic hypotheses to be evaluated with respect to the differing hMSC-EV associated miRNAs. These results align well with the growing understanding that the hMSC plasticity, influenced by inflammatory environmental factors such as pro-inflammatory cytokines, drives alterations in EV content [57,58]. These observations provide a growing understanding of the complex interplay of inflammatory and other pathways involved in hMSCs actions in the lung and provide important information towards developing more effective hMSC-based cell therapies for ARDS and other lung diseases.

## Conclusions

The present study provides evidence that suggests that packaging of miRNAs is influenced by the inflammatory environment in the lungs encountered by the parent hMSCs. EV preparation samples obtained from all hMSCs examined are functional, *i.e.* they reduced CFTR Cl^−^ secretion, an effect predicted to inhibit mucociliary clearance, an effect that is of potential physiologic and clinical significance. While the mechanism(s) involved remain unknown, the current data provide an extensive descriptive platform for targeted mechanistic investigations of specific miRNAs in acute lung injury and development of potential new hMSC-based EV therapeutics to reduce inflammation that leads to significant lung injury and often death. It will be essential to develop EV therapeutics that reduce inflammation and promote lung injury but do not have an untoward effect on mucociliary clearance.

## Supplementary Material

1

2

3

4

5

6

7

8

9

10

11

12

13

14

Supplementary material associated with this article can be found in the online version at doi:10.1016/j.jcyt.2025.01.006.

## Figures and Tables

**Table 1:** Functional predictions—focus on possible roles in acute lung injury.

		HV vs Control		ARDS vs control		ARDS vs HV		Possible roles of importance in acute lung injury	
MiRBase ID	MiRBase accession #	Log2 FC	Adj *P* value	Log2 FC	Adj *P* value	Log2 FC	Adj *P* value	Function based on empirically confirmed targets (TarBase v8.0)	Function based on computationally predicted targets (DIANA - target scan)
hsa-miR-7107-5p	MIMAT0028111	1.03	3.50E-03	2.42	8.82E-13	1.39	2.33E-04	No hits found on TarBase v8.0	No related GO category found
hsa-miR-6803-5p	MIMAT0027506	1.29	2.84E-04	2.74	1.20E-15	1.44	1.75E-04	No hits found on TarBase v8.0	No related GO category found
hsa-miR-6798-5p	MIMAT0027496	1.62	1.66E-05	3.14	5.36E-19	1.52	1.48E-04	No hits found on TarBase v8.0	No related GO category found
hsa-miR-760	MIMAT0004957	1.13	1.79E-03	2.79	5.68E-16	1.66	1.30E-05	Regulates RHO GTPases via IQ motif-containing GTPase-activating proteins (IQGAPs) - scaffolding proteins playing central roles in cell-cell adhesion, polarity, and motility.	Predicted to regulate integrity of adherens junctions and the first step of cholesterol, steroid hormones, and vitamin D biosynthesis (Lanosterol Synthase and 24-Dehydrocholes- terol Reductase)
hsa-miR-6727-5p	MIMAT0027355	1.20	8.04E-04	3.04	2.90E-19	1.84	9.51E-07	No hits found on TarBase v8.0	Predicted Glycosphingolipid biosynthesis (ST8 Alpha-N-Ace- tyl-Neuraminide Alpha-2,8-Sialyltransferase 5) and Phosphatidylinositol signaling system (PIP5K1C, PIK3R2 and PLCB2)
hsa-miR-4763-3p	MIMAT0019913	1.36	1.18E-03	4.41	8.89E-30	3.06	2.00E-13	No hits found on TarBase v8.0	Putative regulation of GPX7 (Glutathione Peroxidase 7) involved in Glutathione metabolism and Detoxification of Reactive Oxygen Species.
hsa-miR-3652	MIMAT0018072	1.02	1.59E-02	4.15	3.33E-25	3.13	1.83E-13	No hits found on TarBase v8.0	No related GO category found
hsa-miR-885-3p	MIMAT0004948	1.20	5.52E-03	4.58	8.89E-30	3.37	2.78E-15	Regulates SERPINE2 (serine protease inhibitor e.g. Thrombin, urokinase, plasmin and trypsin); regulates NFKB Inhibitor Zeta; TNFAIP3; and IRF3	Putative regulation of cell adhesion via ICAM3 and NECTIN1 (organization of adherens junctions and tight junctions in epithelial and endothelial cells)
hsa-miR-766-3p	MIMAT0003888	−1	1.24E-03	−1.6	2.59E-08	−1.4	4.61E-07	Anti-inflammatory role by regulating complement 3 (C3), Jun, IRF3, STAT2, MAPK1, MAPK8, TBK1, TANK (inhibits TRAF)	Predicted to regulate fatty acid synthase (FASN) and Acetyl-CoA carboxylase (ACAC) - rate limiting enzymes in the synthesis of fatty acids.
hsa-miR-3175	MIMAT0015052	N/C	N/C	1.51	4.80E-08	1.2	1.07E-04	Negative regulation of metabolic processes and DNA damage response dependent upon p53 - may impact apoptosis in lung injury	Predicted to regulate vasopressin water resorption (Aqp2 and VAMP2) and ECM-receptor interaction (HSPG2, COL5A1, COL1A1, COL5A3)
hsa-miR-6893-5p	MIMAT0027686	N/C	N/C	1.98	2.77E-15	1.84	1.69E-12	No hits found on TarBase v8.0	Predicted to affect SNARE interactions in vesicular transport
hsa-miR-6875–5p	MIMAT0027650	N/C	N/C	3.24	1.04E-21	3.15	3.45E-19	No hits found on TarBase v8.0	Predicted to regulate proteoglycans, Hyaluron (CD44), Heparan sulfate (SDC2) and fibroblast growth factor receptor 1 (FGFR1) important in wound healing, cell migration and repair.
hsa-miR-6799–5p	MIMAT0027498	N/C	N/C	1.97	6.16E-12	1.92	1.45E-10	No hits found on TarBase v8.0	Predicted to regulate Von Willebrand Factor (vWF) critical for ECM interactions. HACD2 (3-Hydroxyacyl-CoA Dehydratase 2) in the conversion of long chain fatty acids to very long chain fatty acids. Pyridoxamine 5^0^ -Phosphate Oxidase (PNPO) rate-limiting step in the synthesis of pyridoxal 5^0^- phosphate (vitamin B6) - required co-factor in both homocysteine metabolism and synthesis of neurotransmitters such as catecholamines.
hsa-miR-6787–5p	MIMAT0027474	N/C	N/C	2.69	2.90E-16	1.93	5.31E-08	No hits found on TarBase v8.0	Predicted to regulate fatty acid biosynthesis and metabolism through regulation of fatty acid synthase (FASN), Acyl-CoA Synthetase Long Chain Family Member 3 (ACLS3) and Trans-2,3-Enoyl-CoA Reductase (TECR)

miR, microRNA; DE, differential expressed; HV, healthy volunteer; ARDS, Acute Respiratory Distress Syndrome; FC, fold change; Adj, adjusted.

**Table 2:** Most targeted KEGG pathways.

KEGG ID	Total strong interactions	Genes on path	Path name
1100	11555	1117	Metabolic pathways
4010	5614	268	MAPK signaling pathway
5200	5199	326	Pathways in cancer - Homo sapiens (human)
4020	3590	177	Calcium signaling pathway
4650	3547	136	Natural killer cell mediated cytotoxicity
4080	3509	272	Neuroactive ligand-receptor interaction
4144	3461	201	Endocytosis
4510	3421	200	Focal adhesion
4612	3235	76	Antigen processing and presentation
4810	3160	212	Regulation of actin cytoskeleton
4380	2981	128	Osteoclast differentiation
4060	2871	265	Cytokine-cytokine receptor interaction
4310	2706	150	Wnt signaling pathway
4145	2640	152	Phagosome
4514	2501	133	Cell adhesion molecules
4062	2366	189	Chemokine signaling pathway
5010	2348	161	Alzheimer disease
4910	2320	138	Insulin signaling pathway
4360	2297	129	Axon guidance
4722	2245	127	Neurotrophin signaling pathway - Homo sapiens (human)

The 20 pathways predicted to be most highly targeted by 14 miRNAs selected according to the criteria defined in Methods. The first column is the KEGG pathway ID. The second column shows total number of times any transcript of any gene belonging to a to a pathway was strongly targeted based on having an IntaRNA energy score in the bottom quartile of all energy scores predicted for interactions between the 14 miRNA and the human reference transcriptome. The total number of genes on the KEGG pathway and its name are given in the last two columns. Pathways of special interest to the present study are shown in bold.

## Data Availability

Data has been deposited in Gene Expression Omnibus (GEO, GSE282919).

## Glossary

ARDS: Acute respiratory distress syndrome
AUROC: area under the receiver operating characteristic
BALF: bronchoalveolar lavage fluid
CFTR: cystic fibrosis transmembrane conductance regulator
DA: discriminant analysis
DEA: Differential expression analysis
Evs: extracellular vesicles
FDR: False Discovery Rate
HBEC: human airway epithelial cells
hMSC: human bone marrow-derived mesenchymal stromal cells
HV: healthy volunteers
miRNA: microRNA
NTA: nanoparticle tracking analyses
PLS: Sparse Partial Least Squares
PLS-DA: Sparse Partial Least Squares discriminant analysis
SAM: Significance analyses of microarrays
