# Supplementary material for: Acute respiratory distress vs healthy lung environments differently affect mesenchymal stromal cell extracellular vesicle miRNAs
Source: Cytotherapy. Author manuscript; Available in PMC 2026 May 18. (PMC13181138; doi:10.1016/j.jcyt.2025.01.006)
Supplement: 3 [file NIHMS2170204-supplement-3.pptx]

## Slide 1
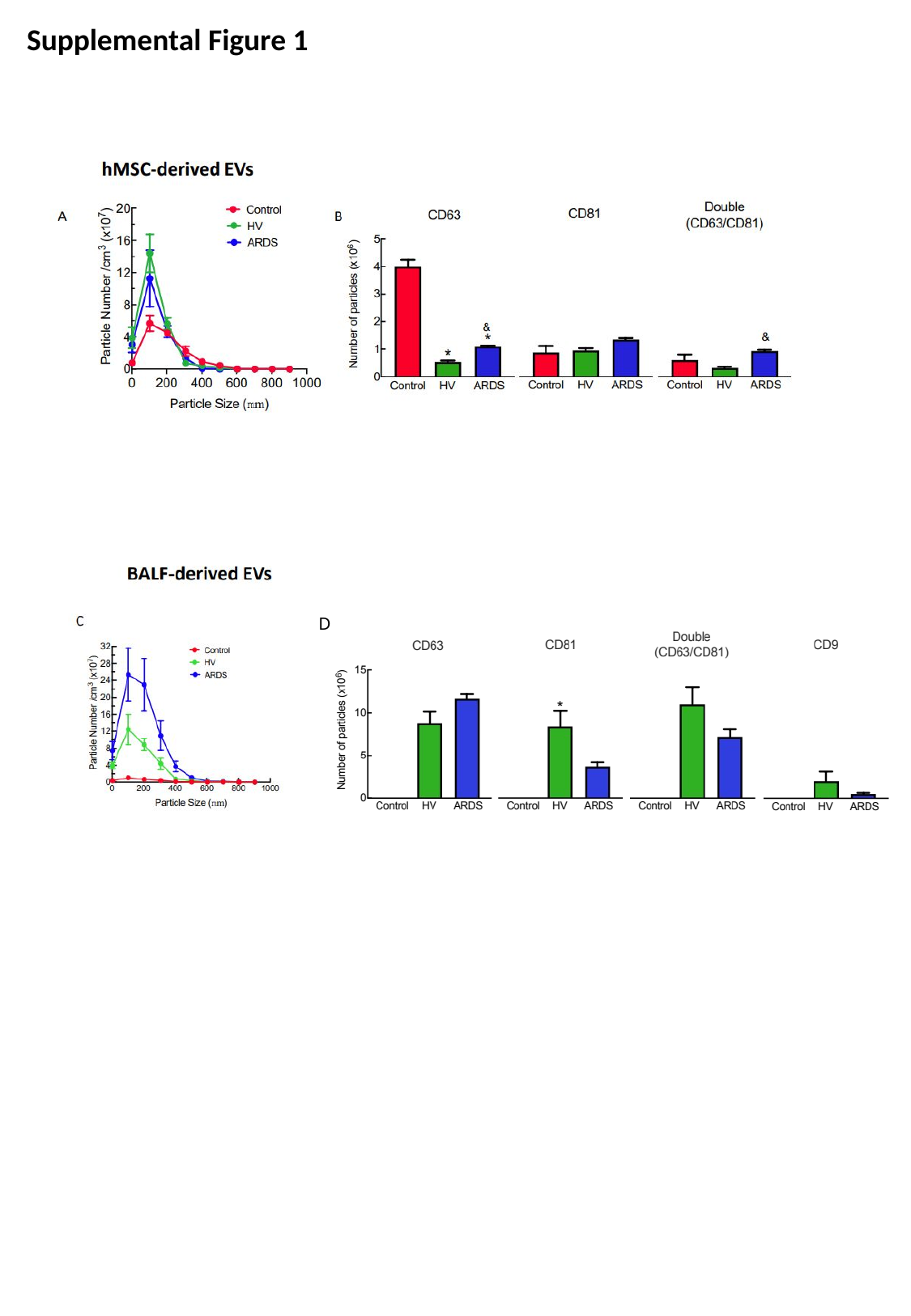

Supplemental Figure 1
D

## Slide 2
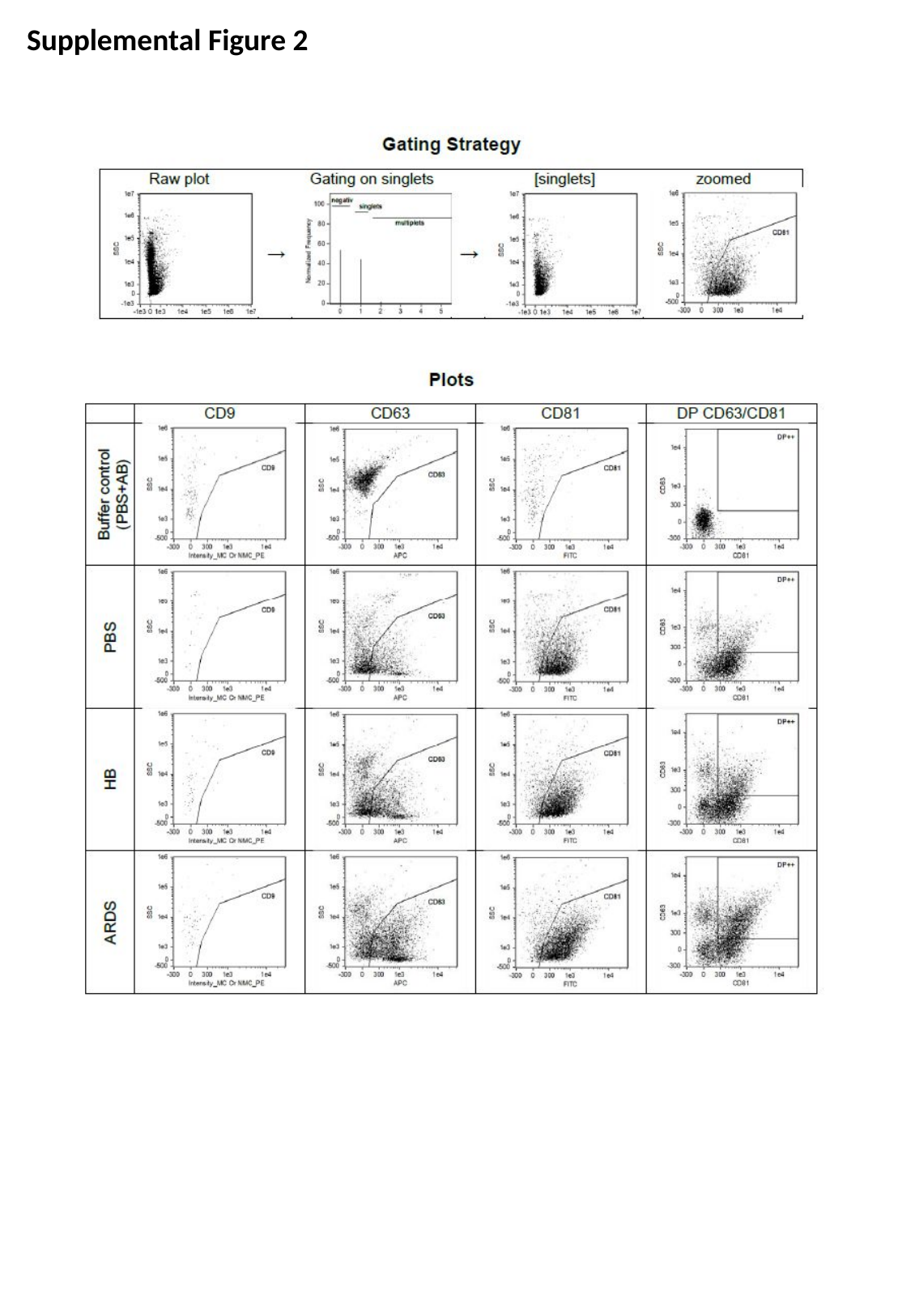

Supplemental Figure 2

## Slide 3
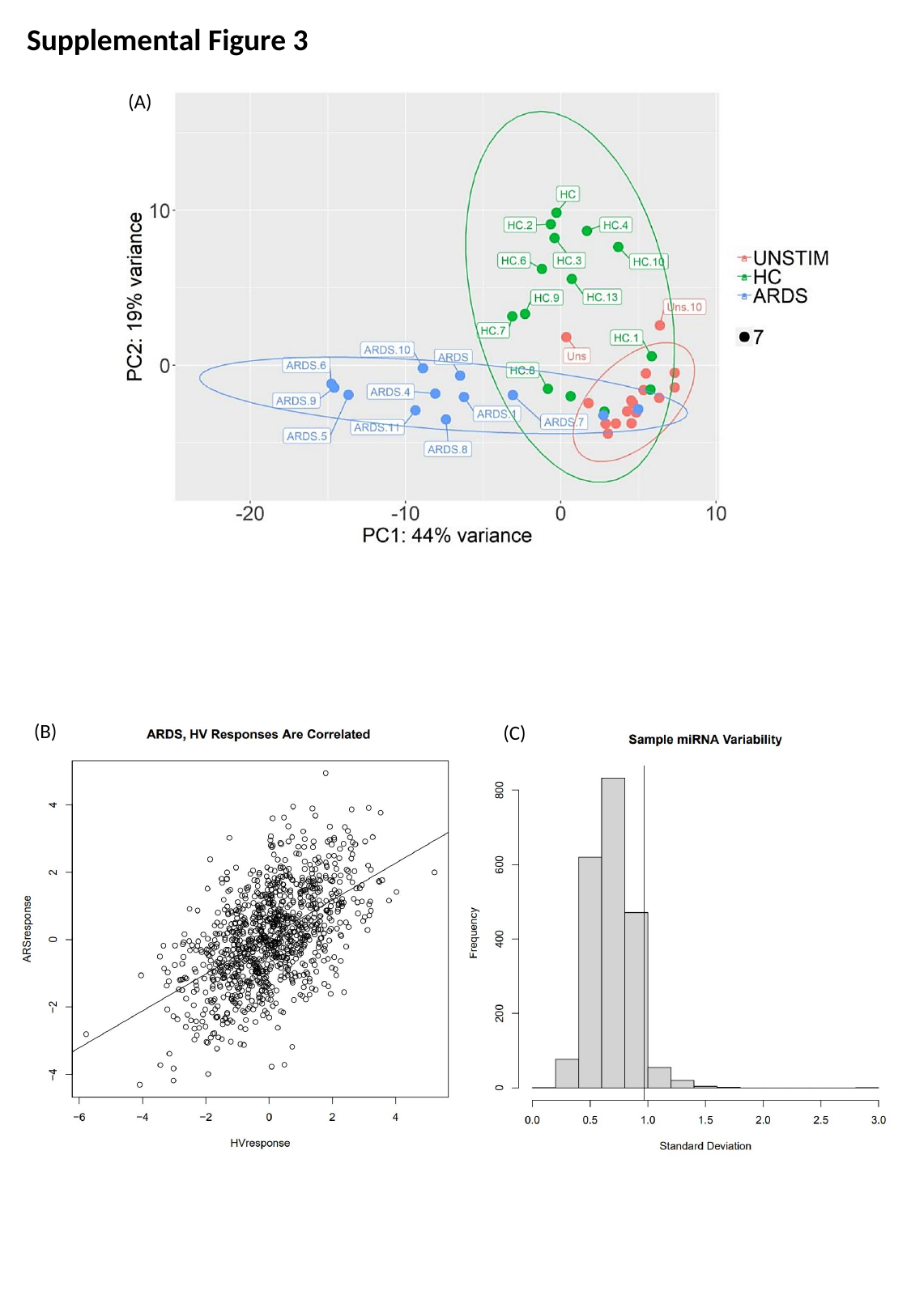

Supplemental Figure 3
(A)
(B)
(C)

## Slide 4
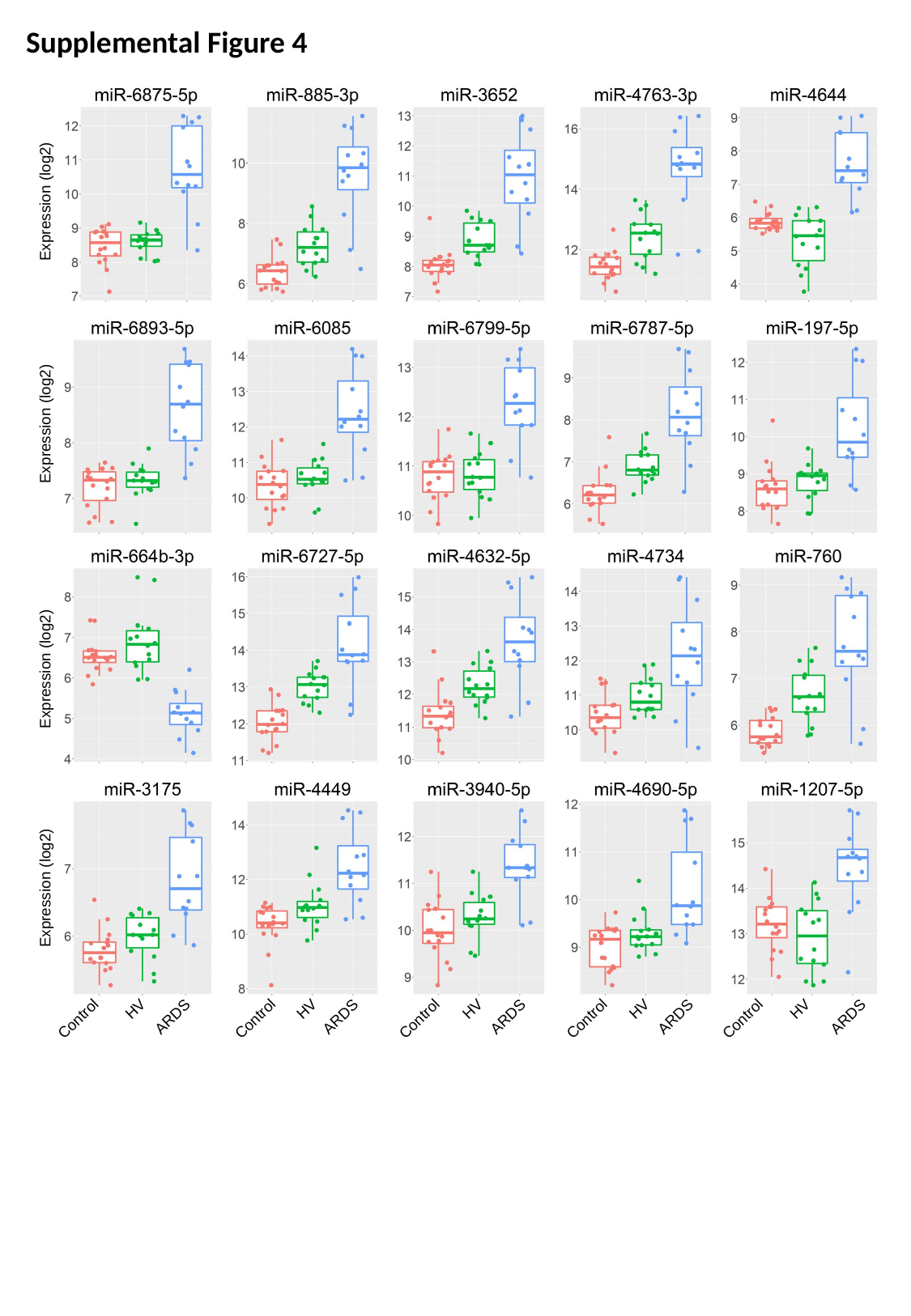

Supplemental Figure 4
Expression (log2)
Expression (log2)
Expression (log2)
Expression (log2)
HV
HV
HV
HV
HV
ARDS
ARDS
ARDS
ARDS
ARDS
Control
Control
Control
Control
Control

## Slide 5
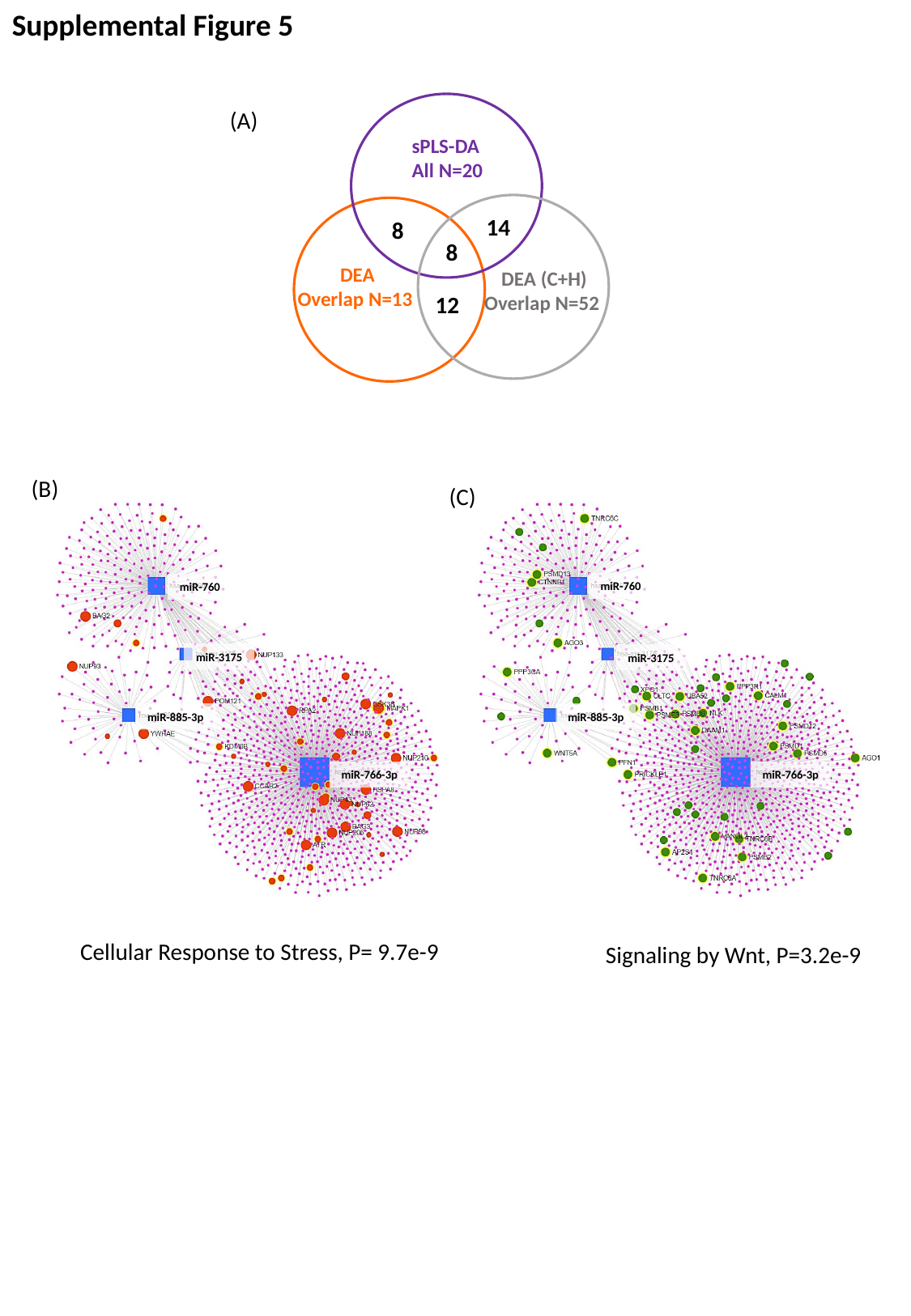

Supplemental Figure 5
(A)
sPLS-DA
All N=20
14
8
8
DEA
Overlap N=13
DEA (C+H)
Overlap N=52
12
(B)
(C)
miR-760
miR-760
miR-3175
miR-3175
miR-885-3p
miR-885-3p
miR-766-3p
miR-766-3p
Cellular Response to Stress, P= 9.7e-9
Signaling by Wnt, P=3.2e-9
