# Supplementary material for: Acute respiratory distress vs healthy lung environments differently affect mesenchymal stromal cell extracellular vesicle miRNAs
Source: Cytotherapy. Author manuscript; Available in PMC 2026 May 18. (PMC13181138; doi:10.1016/j.jcyt.2025.01.006)
Supplement: 4 [file NIHMS2170204-supplement-4.docx]

**DETAILED** **METHODS**

***Human BALF samples***

Collection and processing of BALF samples from healthy volunteers (HVs) and from ARDS patients was done as previously described^1-3^. In brief, HVs underwent standard fiberoptic bronchoscopy of the right middle lobe at Dartmouth-Hitchcock Medical Center (Lebanon, NH) between January and July 2018 under appropriate institutional IRB protocols. Exclusion criteria for HVs were: history of cardiopulmonary disease, regular smoking or vaping, and use of immunomodulatory medications. BALF samples from ARDS patients without sepsis were collected prospectively as part of an unrelated clinical investigation conducted by the National Heart Lung Blood Institute (NHLBI) ARDSNET (ClinicalTrials.gov NCT0011216)^4^. For the HV lavages, 20 ml sterile saline was utilized, and samples were centrifuged and supernatants stored at -70°C. For the ARDS patient lavages, a standard 40 ml mini-BALF with sterile saline was utilized in intubated ARDS patients and BALF samples similarly centrifuged and stored.

***In vitro exposure of hMSCs to BALF***

hMSCs from three healthy donors were obtained from the NHLBI’s Production Assistance for Cellular Therapies (PACT) program (University of Minnesota) and cultured in MEM/EBSS medium supplemented with 1% penicillin/streptomycin and 20% fetal bovine serum in standard tissue culture incubators. The hMSCs have been previously characterized according to criteria from the International Society for Cell and Gene Therapy ^1,5,6^. hMSCs were utilized at passages 3-5 and were the same as those used in a recent trial of hMSC administration in non-COVID ARDS patients [NCT01775774, ^5,6^] and in our previous investigations of the effects of BALF on hMSC gene and protein expression ^1-3^.

hMSCs were seeded into 6-well plates (Corning, 2 x 10^5^ cells/well, 2 wells/BALF sample or control) in cell culture conditions outlined above and incubated overnight. The next day, cells were washed twice with PBS and synchronized for 24 hours in serum-free medium. After synchronization, the serum-free medium was replaced with 1 ml of serum-free medium containing either individual ARDS (N=16) or individual HV (N=16) BALF samples at a 20% (v/v) concentration ^1-3^. Control hMSCs were exposed to serum-free medium only (N=16). After 5 hours incubation at 37°C in a standard tissue culture incubator, cell culture medium was removed, cells were washed once with PBS, and 2 ml serum-free medium added per well. After 48 hours incubation (37°C), the conditioned medium was collected, passed through a 0.8 μm syringe filter, and processed for EV preparation, NTA, and miRNA assessments, as described below.

***Extracellular vesicles (EVs) preparation and miRNA isolation***

EVs obtained either from hMSCs exposed to serum-free medium or to individual HV or ARDS BALF samples or directly from pure BALF samples obtained from HVs (N=4) and ARDS patients (N=4). were prepared in accordance with recent recommendations from the International Society for Extracellular Vesicles (ISEV) ^7^. EV and EV-derived miRNAs were prepared from the conditioned medium and BALF samples using the exoRNeasy Serum/Plasma Maxi/Maxi Midi Kit (Qiagen, Germantown, MD, USA), according to manufacturer’s instructions. Sample preparations were stored at -20ºC until miRNA sequencing was performed. For nanoparticle tracking analysis (NTA) and imaging flow cytometry analysis, EVs were isolated using ExoQuick-TC (cat # EXOTC50A; System Biosciences, Palo Alto, CA) according to the manufacturer’s protocol.

***NTA***

NTA (ZetaView, Particle Metrix Inc, Germany 405 nm/488 dual excitation lasers) was used to measure size and concentration of particles in EV preparations. Video acquisition was performed with fixed settings for all samples (25°C fixed temperature, 11 positions, 2 cycles, sensitivity 84, Shutter 100, 30 frame rate per second, and 3-6 measurements). Videos of all 11 positions were recorded for each sample with 5 cycles (1 cycle equals 1s) at each position and analyzed with the ZetaView analysis software (Version 8.05.11).

***Imaging Flow Cytometry***

To assess the presence of tetraspanin (CD9, CD63, and CD81) positive objects, predominantly EVs, 2 x 10^6^ cells (for each condition) were exposed for 5 hours at 37°C to either ARDS or HV BALF samples or to serum-free medium (control). Given the large BALF volume (20% v/v) required for exposures and limited amounts of available BALF, pooled rather than individual HV or ARDS samples were used for these studies. Imaging flow cytometry was performed on the AMNIS ImageStreamX Mark II Flow Cytometer (AMNIS/Luminex, Seattle, WA, USA) as previously described ^8,9^. Details for all antibodies used are provided in **Supplementary Table 11**. In brief, antibodies were added to the samples and incubated for 1 hour at room temperature. According to the MIFlowCyt-EV guidelines ^10^, controls included EV samples without antibodies (unlabeled, uEVs), NaCl-HEPES buffer with antibodies but without EV samples, as well as antibody labeled EV samples exposed to 1% NP40 (Calbiochem, San Diego, CA, USA). After labeling without any washing, samples were diluted with PBS and analyzed using the built-in autosampler for 96-well round bottom plates. Acquisition time was selected as 5 minutes per well. Data were acquired at 60x magnification, low flow rate, and with the removed beads option deactivated. Data were analyzed as described previously with the IDEAS software (version 6.2) ^8,9^. Fluorescent events were plotted against the side scatter (SSC). A combined mask feature was used (MC and NMC) to improve the detection of fluorescent images. Images were analyzed for coincidences (swarm detection) by using the spot counting feature. Every data point with multiple objects was excluded from the analyses. Objects with low side scatter values (< 500) and fluorescence intensities higher than 300 were considered as small EVs (sEVs). Average concentrations were calculated according to the acquisition volume and time.

*NTA and Image Flow Analyses data*

Particle sizes and counts obtained with ZetaView were plotted in 100 νm bins on an X-axis ranging between 2.5-903 μm and the area under the curve (AUC) for each sample was calculated. Data were analyzed using one-way ANOVA, with group (control, HV, ARDS) as a factor and Bonferroni post-hoc correction tests were done to identify differences between the group means. The number of positively stained objects for each tetraspanin antibody (CD9, CD63, and CD81), as well as the number of double positive (CD63/CD81) were similarly analyzed within each tetraspanin type. Differences in positively stained objects between HV and ARDS BALF samples were tested using unpaired t-test. All analyses and graphs were done using Prism (version 9.3, GraphPad software).

***MiRNA sequencing and Differential Expression Analysis***

RNA was isolated from each EV preparation as described above and only samples that passed quality control with an A260/A280 above 1.80 were used for RNA sequencing (control=16; ARDS=12; HV=14). A total of 35 ul (35 ng/µl) of eRNA (EV preparation-derived RNA) was used for miRNA sequencing performed using the HTG EdgeSeq miRNA Whole Transcriptome Assay (miRNA WTA, as per manufacturer’s instructions as published). The miRNA library was sequenced on a NextSeq (Illumina, Inc., San Diego, CA) using a V3 150-cycle kit with two index reads. PhiX (Roche, Mississauga, ON, CAN). Data were returned from the sequencer in the form of demultiplexed FASTQ files, with one file per original well of the assay. The HTG EdgeSeq Parser (v. 5.0.535.3181, HTG Molecular, Tucson, AZ, USA) was used to align the FASTQ files to the probe list to collate the data. Raw read counts for the 42 samples were imported into R to perform differential expression analyses with the R package DESeq2 ^11^. Variance stabilization transformation was used to prepare data for analyses. Significance analyses of microarrays (SAM) was ran using one class analysis approach to identify miRNAs over-represented in EVs derived from control hMSCs ^12^ based on the normalized data using Variance stabilization transformation. The delta value was set to 7 (the best delta parameter value selected by the software with the lowest False Discovery Rate (FDR]) using 1000 permutations, FDR cut-off was 0 (%).

Target prediction and functional analyses were conducted using miRNet ([https://www.mirnet.ca](about:blank)) ^13^. Enriched Reactome pathways were selected by hypergeometric tests of miRNAs having FDR ≥0.05. Putative relationships were obtained using DIANA-TarBase v8 (collection of experimentally supported miRNA–gene interactions) ^14^.

***Supervised classification analysis for ARDS, HV, and control hMSC EV miRNAs***

Sparse Partial Least Squares (PLS) discriminant analysis (sPLS-DA) (R package “mixOmics”) was performed to identify and narrow the list of miRs regulated by ARDS exposure**,** using a different analysis approach, and narrow the miRs to those that were not only differentially expressed but also able to discriminate between treatment groups. The area under the receiver operating characteristic (AUROC) curve for all-vs-one comparisons for PLS-DA are based on predicted maximum distances averaged over all cross-validations and complement the analysis rather than evaluate model performance.

***In silico target predictions for transcripts, genes and pathways***

IntaRNA ^15^ was used to predict the most likely targets of the top 14 miRNA selected to be different between the various treatments to extend miRNet Tarbase predictions considering 10 of the newly identified miRNAs were found not to have experimentally proven targets. Interactions between miRNA and our reference transcriptome (https://bio.tools/intarna) with energy scores in the bottom quartile, i.e., an energy score less than -20.7, were categorized as strong predicted targeting to focus on predicted targeting interactions most likely to degrade message or interfere with translation. To estimate the overall targeting of a gene by the 14 miRNAs collectively, we assumed a simple additive model and counted the number of times each transcript of a gene was strongly targeted by any of the 14 miRNAs. Interference at the pathway level was estimated as the total number of strong targeting interactions for all the KEGG genes on a given pathway identified by the KEGGREST R package ^16^. Finally, the most strongly targeted pathways relevant to ARDS were visualized using the R package pathview (https://pathview.uncc.edu/home).

***Human bronchial epithelial cells***

### Primary wild-type human airway epithelial cells (HBEC) were obtained from Dr. Scott Randell (University of North Carolina, Chapel Hill, NC) and cultured as previously described ^17^. The Dartmouth Committee for the Protection of Human Subjects determined that the use of HBEC in this study is not considered human subject research because cells are taken from discarded tissue and contain no patient identifiers. Briefly, HBEC from passages 4 and 5 were grown in standard cell culture conditions (37 °C, 5% CO_2_) in BronchiaLife basal medium (Lifeline Cell Technology, Frederick, MD) supplemented with the BronchiaLife B/T LifeFactors Kit (Lifeline) as well as 10,000 U/mL penicillin and 10,000 μg/mL streptomycin (Sigma-Aldrich, St. Louis, MO). Results were independent of passage number. The absence of mycoplasma was verified by routine analysis. HBEC with a viability of 96%–98% were seeded at 2 × 10^6^ cells per T175 cell culture flask and grown to confluence while changing the growth medium every 2–3 days. For measurements of CFTR Cl^-^ secretion and analysis of cytokine and chemokine secretion, HBEC were seeded at 500,000 onto 12-mm Snapwell permeable supports (Corning, Corning, NY) coated with 50 μg/ml Collagen type IV (Sigma-Aldrich, St. Louis, MO) and grown in an air-liquid interface media (ALI) at 37°C for 3–4 weeks to establish polarized monolayers, as described previously ^18,19^.

### ***Cytokine secretion by HBEC***

To assess the ability of hMSC EVs to alter cytokine secretion by HBEC, EVs (2 x 10^7^, a value similar to the concentration observed in BALF and other biological fluids ^20-25^ or an equal volume of process control (PC: media not exposed to HBEC and run through the EV isolation procedure) were added to the apical side of HBEC for 6 hours and then removed. Basolateral media was collected at the 6-hour and 24-hour time points from the same HBEC monolayers used for measurements of CFTR Cl^-^ secretion. Secreted cytokines were measured using the MILLIPLEX MAP Human Cytokine/Chemokine 48-Plex cytokine assay (Millipore). The assays were carried out by the Immune Monitoring, and Flow Cytometry Shared Resource at the Cancer Center at Dartmouth, with NCI Cancer Center Support (P30 CA023108).

#### **Measurements of CFTR Cl^-^ currents**

As described in detail elsewhere ^18,25^, cells on Snapwell filters were mounted in Ussing chambers whereupon the transepithelial voltage was clamped to 0 mV, and the short circuit current (I_sc_) was measured as described previously ^18,25^. Subsequently, amiloride (50 μM) was added to the apical solution to inhibit sodium reabsorption. Thereafter, CFTR Cl^−^ secretion was stimulated with forskolin (10 μM; Sigma-Aldrich), followed by thiazolidinone (CFTR_inh_-172, 20 μM; Millipore, Billerica, MA) an inhibitor of CFTR Cl^−^ secretion.

***Statistical Analyses***

*NTA and Image Flow Analyses data*

Particle sizes and counts obtained with ZetaView were plotted in 100 νm bins on an X-axis ranging between 2.5-903 μm and the area under the curve (AUC) for each sample was calculated. Data were analyzed using one-way ANOVA, with group (control, HV, ARDS) as a factor and Bonferroni post-hoc correction tests were done to identify differences between the group means. The number of positively stained objects for each tetraspanin antibody (CD9, CD63, and CD81), as well as the number of double positive (CD63/CD81) were similarly analyzed within each tetraspanin type. Differences in positively stained objects between HV and ARDS BALF samples were tested using unpaired t-test. All analyses and graphs were done using Prism (version 9.3, GraphPad software).

*miRNA data*

To determine differential expression of miRNAs found in BALF-derived EV and hMSC-derived EV preparations, unpaired T-tests with Welch correction (does not assume equal standard deviations) were performed for those that passed a test for normality (Kolmogorov–Smirnov). For those that were not normally distributed, two-tailed Mann-Whitney tests were performed on a miRNA-by-miRNA basis (p=0.05). Statistical analyses were performed using GraphPad Prism software. The Mann-Whitney test was used to assess differences between groups (comparing top 20 differentially expressed miRs detected in EVs with those present in BALF from patients). P-values ≤0.05 were considered as significant, except in the case of RNA sequencing data analyzed in DESeq2, where a multiple hypothesis corrected FDR less than 0.05 was significant. Spearman correlations were calculated in base R, using the t distribution to calculate P-values in those cases that included ties in rank.

**SUPPLEMENTAL FIGURE LEGENDS**

**Supplemental Figure 1. EV preparations from BALF stimulated hMSCs differ in size distribution and tetraspanin expression compared to EV preparation isolated directly from BALF. (A)** EVs secreted by hMSCs exposed to serum-free medium alone (control) or to BALF collected from HVs or patients with ARDS are similar in size (50-200 μm) and number (n = 3, 4, and 4, for control, HV and ARDS, respectively; please see **Table 1** for specific BALF samples used). Data for each condition are means + SEM per 100 nm bin, as detailed in the methods. AUC for each condition is not statistically different, p>0.05. **(B)** Image flow cytometry of the same EV preparation samples characterized in (**A**) shows both HV and ARDS BALF exposure significantly decreased CD63 positive EVs overall compared to control, with HV BALF exposure resulting in significant lower CD63 expression than ARDS BALF exposure. No significant difference in CD81 expression between groups and no CD9 expression was detected in any group. * Different than control and ^&^ different than HV. **(C)** EV preparation samples present in BALF alone vs control (serum-free medium) show a different pattern than EV preparation samples obtained from BALF-stimulated hMSCs: EV preparation samples of larger size are more prevalent and ARDS BALF has a higher number of EV preparation samples (n=6, 3, 3 for control, HV and ARDS, respectively; please see **Table 1** for specific BALF samples used). Data for each condition are means + SEM per 100 μm bin, as detailed in the methods. The AUC for ARDS is significantly higher than HV and control, p<0.05. **(D)** Image flow cytometry of the same EV preparation samples characterized in (**C**) shows presence of CD9 in addition to CD63 and CD81. EVs present in HV BALF samples expressed significantly increased expression of CD81 compared to ARDS BALF. Statistical analyses were performed by unpaired t-test between HV and ARDS. Abbreviations: ARDS, acute respiratory distress syndrome; AUC, Area Under the Curve; EVs, extracellular vesicles; HV, healthy volunteer; NTA, Nanotracking analysis.

**Supplemental Figure 2.** Gating strategies and individual scattergrams for flow cytometric analyses.

**Supplementary Figure 3: Clustering, correlation, and variability of EV preparation samples-miRNAs (A)** Principal component analysis showing clustering of EV- EV preparation samples miRNAs by treatment**. (B**) Correlation of fold change in count number between miRNAs enriched in the response to ARDS vs HV. **(C)** Variability in the change in mRNA expression (count number=frequency) and the standard deviation across all replicates**.** The top 14 EV preparation sample-miRNAs selected are in the top quartile of most altered miRNAs.

**Supplementary Figure 4. Top 20 (from 52) hMSC-derived EV preparation samples miRNAs demonstrate different expression patterns following HV or ARDS BALF exposure.** Box plots showing change in normalized miRNA counts (Log2) in EV preparation samples derived from control hMSCs (red), treated with BALF from HVs (green) and ARDS patients (blue). Line in box is the median quartile, squares are upper and lower quartiles and whiskers are maximum and minimum range.

**Supplementary Figure 5.** **Top Interaction Network for 14 Differentially Expressed classifiers.** **(A**) A total of 14 of the 20 classifiers identified using sPLS-DA were found to also be differentially expressed between treatment groups. Functional analysis and visual exploration of miRNA–target interactions in miRNet found that 4 of the 14 differentially expressed classifiers form a putative in-silico regulatory network acting as putative “hub” regulators for 1259 putative targets of which 75 are involved in (**B**) the cellular response to stress (red circles) including HSF-1 mediated response to stress (1.51e-7) and (**C**) 74 signaling by Wnt (green circles), specifically beta-catenin independent Wnt signaling (1.1e-6). MiRNA 766-3p, miRNA-760, miR885-3p, and miRNA-3175 are shown as blue squares. Genes in the network but not linked to the Cellular Response to Stress or in Signaling by Wnt are shown as small pink circles. Edges are in gray and describe direct interactions between nodes.

**SUPPLEMENTAL REFERENCES**

1. Abreu, S.C., Rolandsson Enes, S., Dearborn, J., Goodwin, M., Coffey, A., Borg, Z.D., Dos Santos, C.C., Wargo, M.J., Cruz, F.F., Loi, R., DeSarno, M., et al. (2019). Lung inflammatory environments differentially alter mesenchymal stromal cell behavior. Am J Physiol Lung Cell Mol Physiol *317*, L823-L831. 10.1152/ajplung.00263.2019.

2. Abreu, S.C., Hampton, T.H., Hoffman, E., Dearborn, J., Ashare, A., Singh Sidhu, K., Matthews, D.E., McKenna, D.H., Amiel, E., Barua, J., Krasnodembskaya, A., et al. (2020). Differential effects of the cystic fibrosis lung inflammatory environment on mesenchymal stromal cells. Am J Physiol Lung Cell Mol Physiol. 10.1152/ajplung.00218.2020.

3. Rolandsson Enes, S., Hampton, T.H., Barua, J., McKenna, D.H., Dos Santos, C.C., Amiel, E., Ashare, A., Liu, K.D., Krasnodembskaya, A.D., English, K., Stanton, B.A., et al. (2021). Healthy versus inflamed lung environments differentially affect mesenchymal stromal cells. Eur Respir J *58*. 10.1183/13993003.04149-2020.

4. Liu, K.D., Levitt, J., Zhuo, H., Kallet, R.H., Brady, S., Steingrub, J., Tidswell, M., Siegel, M.D., Soto, G., Peterson, M.W., Chesnutt, M.S., et al. (2008). Randomized clinical trial of activated protein C for the treatment of acute lung injury. Am J Respir Crit Care Med *178*, 618-623. 10.1164/rccm.200803-419OC.

5. Matthay, M.A., Calfee, C.S., Zhuo, H., Thompson, B.T., Wilson, J.G., Levitt, J.E., Rogers, A.J., Gotts, J.E., Wiener-Kronish, J.P., Bajwa, E.K., Donahoe, M.P., et al. (2019). Treatment with allogeneic mesenchymal stromal cells for moderate to severe acute respiratory distress syndrome (START study): a randomised phase 2a safety trial. Lancet Respir Med *7*, 154-162. 10.1016/S2213-2600(18)30418-1.

6. Wilson, J.G., Liu, K.D., Zhuo, H., Caballero, L., McMillan, M., Fang, X., Cosgrove, K., Vojnik, R., Calfee, C.S., Lee, J.W., Rogers, A.J., et al. (2015). Mesenchymal stem (stromal) cells for treatment of ARDS: a phase 1 clinical trial. Lancet Respir Med *3*, 24-32. 10.1016/S2213-2600(14)70291-7.

7. Thery, C., Witwer, K.W., Aikawa, E., Alcaraz, M.J., Anderson, J.D., Andriantsitohaina, R., Antoniou, A., Arab, T., Archer, F., Atkin-Smith, G.K., Ayre, D.C., et al. (2018). Minimal information for studies of extracellular vesicles 2018 (MISEV2018): a position statement of the International Society for Extracellular Vesicles and update of the MISEV2014 guidelines. J Extracell Vesicles *7*, 1535750. 10.1080/20013078.2018.1535750.

8. Tertel, T., Gorgens, A., and Giebel, B. (2020). Analysis of individual extracellular vesicles by imaging flow cytometry. Methods Enzymol *645*, 55-78. 10.1016/bs.mie.2020.05.013.

9. Tertel, T., Bremer, M., Maire, C., Lamszus, K., Peine, S., Jawad, R., Andaloussi, S.E.L., Giebel, B., Ricklefs, F.L., and Gorgens, A. (2020). High-Resolution Imaging Flow Cytometry Reveals Impact of Incubation Temperature on Labeling of Extracellular Vesicles with Antibodies. Cytometry A *97*, 602-609. 10.1002/cyto.a.24034.

10. Welsh, J.A., Van Der Pol, E., Arkesteijn, G.J.A., Bremer, M., Brisson, A., Coumans, F., Dignat-George, F., Duggan, E., Ghiran, I., Giebel, B., Gorgens, A., et al. (2020). MIFlowCyt-EV: a framework for standardized reporting of extracellular vesicle flow cytometry experiments. J Extracell Vesicles *9*, 1713526. 10.1080/20013078.2020.1713526.

11. Love, M.I., Huber, W., and Anders, S. (2014). Moderated estimation of fold change and dispersion for RNA-seq data with DESeq2. Genome Biol *15*, 550. 10.1186/s13059-014-0550-8.

12. Tusher, V.G., Tibshirani, R., and Chu, G. (2001). Significance analysis of microarrays applied to the ionizing radiation response. Proc Natl Acad Sci U S A *98*, 5116-5121. 10.1073/pnas.091062498.

13. Chang, L., Zhou, G., Soufan, O., and Xia, J. (2020). miRNet 2.0: network-based visual analytics for miRNA functional analysis and systems biology. Nucleic Acids Res *48*, W244-W251. 10.1093/nar/gkaa467.

14. Karagkouni, D., Paraskevopoulou, M.D., Chatzopoulos, S., Vlachos, I.S., Tastsoglou, S., Kanellos, I., Papadimitriou, D., Kavakiotis, I., Maniou, S., Skoufos, G., Vergoulis, T., et al. (2018). DIANA-TarBase v8: a decade-long collection of experimentally supported miRNA-gene interactions. Nucleic Acids Res *46*, D239-D245. 10.1093/nar/gkx1141.

15. D, T. (2019). Suggests RUnit, Maintainer Bioconductor Package Maintainer, Marc Carlson, Pathways biocViews Annotation, and K. E. G. G. ThirdPartyClient. "Package ‘KEGGREST’." R Foundation for Statistical Computing. R Foundation for Statistical Computing: Vienna, Austria

16. Dos Santos, C.C., Amatullah, H., Vaswani, C.M., Maron-Gutierrez, T., Kim, M., Mei, S.H.J., Szaszi, K., Monteiro, A.P.T., Varkouhi, A.K., Herreroz, R., Lorente, J.A., et al. (2022). Mesenchymal stromal (stem) cell therapy modulates miR-193b-5p expression to attenuate sepsis-induced acute lung injury. Eur Respir J *59*. 10.1183/13993003.04216-2020.

17. Fulcher, M.L., and Randell, S.H. (2013). Human nasal and tracheo-bronchial respiratory epithelial cell culture. Methods Mol Biol *945*, 109-121. 10.1007/978-1-62703-125-7_8.

18. Stanton, B.A., Coutermarsh, B., Barnaby, R., and Hogan, D. (2015). Pseudomonas aeruginosa Reduces VX-809 Stimulated F508del-CFTR Chloride Secretion by Airway Epithelial Cells. PLoS One *10*, e0127742. 10.1371/journal.pone.0127742.

19. Swiatecka-Urban, A., Moreau-Marquis, S., Maceachran, D.P., Connolly, J.P., Stanton, C.R., Su, J.R., Barnaby, R., O'Toole G, A., and Stanton, B.A. (2006). Pseudomonas aeruginosa inhibits endocytic recycling of CFTR in polarized human airway epithelial cells. Am J Physiol Cell Physiol *290*, C862-872. 10.1152/ajpcell.00108.2005.

20. Nirujogi, T.S., Kotha, S.R., Chung, S., Reader, B.F., Yenigalla, A., Zhang, L., Shapiro, J.P., Wisler, J., Christman, J.W., Maddipati, K., Parinandi, N.L., et al. (2022). Lipidomic Profiling of Bronchoalveolar Lavage Fluid Extracellular Vesicles Indicates Their Involvement in Lipopolysaccharide-Induced Acute Lung Injury. J Innate Immun *14*, 555-568. 10.1159/000522338.

21. Johnsen, K.B., Gudbergsson, J.M., Andresen, T.L., and Simonsen, J.B. (2019). What is the blood concentration of extracellular vesicles? Implications for the use of extracellular vesicles as blood-borne biomarkers of cancer. Biochim Biophys Acta Rev Cancer *1871*, 109-116. 10.1016/j.bbcan.2018.11.006.

22. Liu, H., Yuan, W., Pang, Q., Xue, C., and Yan, X. (2022). Single-particle analysis of tear fluid reveals abundant presence of tissue factor-exposing extracellular vesicles with strong coagulation activity. Talanta *239*, 123089. 10.1016/j.talanta.2021.123089.

23. Kupsco, A., Prada, D., Valvi, D., Hu, L., Petersen, M.S., Coull, B., Grandjean, P., Weihe, P., and Baccarelli, A.A. (2021). Human milk extracellular vesicle miRNA expression and associations with maternal characteristics in a population-based cohort from the Faroe Islands. Sci Rep *11*, 5840. 10.1038/s41598-021-84809-2.

24. Rahat, S.T., Makela, M., Nasserinejad, M., Ikaheimo, T.M., Hyrkas-Palmu, H., Valtonen, R.I.P., Roning, J., Sebert, S., Nieminen, A.I., Ali, N., and Vainio, S. (2023). Clinical-Grade Patches as a Medium for Enrichment of Sweat-Extracellular Vesicles and Facilitating Their Metabolic Analysis. Int J Mol Sci *24*. 10.3390/ijms24087507.

25. Stanton, B.A. (2017). Effects of Pseudomonas aeruginosa on CFTR chloride secretion and the host immune response. Am J Physiol Cell Physiol *312*, C357-C366. 10.1152/ajpcell.00373.2016.
