# Supplementary material for: Acute respiratory distress vs healthy lung environments differently affect mesenchymal stromal cell extracellular vesicle miRNAs
Source: Cytotherapy. Author manuscript; Available in PMC 2026 May 18. (PMC13181138; doi:10.1016/j.jcyt.2025.01.006)
Supplement: 5 [file NIHMS2170204-supplement-5.docx]

| Analyte | HC BALF | | ARDS BALF | | Control | | PC | |
| --- | --- | --- | --- | --- | --- | --- | --- | --- |
|  | *6hr* | *24hr* | *6hr* | *24hr* | *6hr* | *24hr* | *6hr* | *6hr* |
| G-CSF | 267 | 387 | 315 | 690 | 687 | 547 | 1001 | 529 |
| GROa | 5137 | 6193 | 5297 | 6718 | 7662 | 6676 | 8161 | 5045 |
| IFNy | 11 | 11 | 12 | 12 | 14 | 11 | 16 | 12 |
| IL-6 | 764 | 1176 | 945 | 1691 | 1325 | 1458 | 1484 | 2130 |
| M-CSF | 1070 | 1485 | 1526 | 1964 | 1491 | 1777 | 1363 | 1883 |
| VEGF-A | 259 | 227 | 269 | 285 | 268 | 267 | 369 | 252 |

Analyte concentrations (picograms/ml) are presented as the mean of three replicates per donor, and two different donor were used. Abbreviations: HC, Healthy control; ARDS, acute respiratory distress syndrome; BALF, bronchoalveolar lavage fluid; Control, EVs secreted from hMSCs exposed to serum free medium; PC, process control; EVs, extra cellular vecsicles; HBECs, primary human bronchial epithelial cells; hr, hours; G-CSF, Granulocyte colony-stimulating factor; GROa, chemokine (C-X-C motif) ligand 1; IFNy, interferon gamma; IL-6, interleukin 6; M-CSF, macrophage colony-stimulating factor; VEGF-A, vascular endothelial growth factor A.

**Supplemental Table 8. Cytokines measured in ALI cultured HBECs exposed to EVs**
