# Supplementary material for: Acute respiratory distress vs healthy lung environments differently affect mesenchymal stromal cell extracellular vesicle miRNAs
Source: Cytotherapy. Author manuscript; Available in PMC 2026 May 18. (PMC13181138; doi:10.1016/j.jcyt.2025.01.006)
Supplement: 6 [file NIHMS2170204-supplement-6.docx]

| **Supplementary Table 16. List of antibodies used for EV characterization** | | | | |
| --- | --- | --- | --- | --- |
| ***Antigen*** | ***Conjugate*** | ***Host/isotype*** | ***Clone*** | ***Supplier*** |
| **Human CD9** | PE | Mouse, IgG1 | MEM-61 | EXBIO |
| **Human CD63** | APC | Mouse, IgG1 | MEM-259 | EXBIO |
| **Human CD81** | FITC | Mouse, IgG2a | JS64 | Beckman-Coulter |
